# Advancements in Phosphodiesterase 5 Inhibitors: Unveiling Present and Future Perspectives

**DOI:** 10.3390/ph16091266

**Published:** 2023-09-06

**Authors:** Ahmed K. ElHady, Dalia S. El-Gamil, Mohammad Abdel-Halim, Ashraf H. Abadi

**Affiliations:** 1 School of Life and Medical Sciences, University of Hertfordshire Hosted by Global Academic Foundation, New Administrative Capital, Cairo 11865, Egypt; a.kamal@gaf.edu.eg; 2 Department of Chemistry, Faculty of Pharmacy, Ahram Canadian University, Cairo 12451, Egypt; daliashgamil@gmail.com; 3 Department of Pharmaceutical Chemistry, Faculty of Pharmacy and Biotechnology, German University in Cairo, Cairo 11835, Egypt; mohammad.abdel-halim@guc.edu.eg

**Keywords:** phosphodiesterase 5 inhibitors, selectivity, NO/cGMP, erectile dysfunction, pulmonary arterial hypertension

## Abstract

Phosphodiesterase 5 (PDE5) inhibitors presented themselves as important players in the nitric oxide/cGMP pathway, thus exerting a profound impact on various physiological and pathological processes. Beyond their well-known efficacy in treating male erectile dysfunction (ED) and pulmonary arterial hypertension (PAH), a plethora of studies have unveiled their significance in the treatment of a myriad of other diseases, including cognitive functions, heart failure, multiple drug resistance in cancer therapy, immune diseases, systemic sclerosis and others. This comprehensive review aims to provide an updated assessment of the crucial role played by PDE5 inhibitors (PDE5-Is) as disease-modifying agents taking their limiting side effects into consideration. From a medicinal chemistry and drug discovery perspective, the published PDE5-Is over the last 10 years and their binding characteristics are systemically discussed, and advancement in properties is exposed. A persistent challenge encountered with these agents lies in their limited isozyme selectivity; considering this obstacle, this review also highlights the breakthrough development of the recently reported PDE5 allosteric inhibitors, which exhibit an unparalleled level of selectivity that was rarely achievable by competitive inhibitors. The implications and potential impact of these novel allosteric inhibitors are meticulously explored. Additionally, the concept of multi-targeted ligands is critically evaluated in relation to PDE5-Is by inspecting the broader spectrum of their molecular interactions and effects. The objective of this review is to provide insight into the design of potent, selective PDE5-Is and an overview of their biological function, limitations, challenges, therapeutic potentials, undergoing clinical trials, future prospects and emerging uses, thus guiding upcoming endeavors in both academia and industry within this domain.

## 1. Introduction

PDE5 inhibitors (PDE5-Is) are groundbreaking medications for treating ED. They increase cGMP levels, causing muscle relaxation and vasodilation in the penis, leading to erections. Their therapeutic potential extends beyond ED, with clinical approval for treating PAH, BPH, and LUTS. Research highlights their potential in diseases like cancer, neurological disorders, cystic fibrosis, and diabetes. Promisingly, this has incited the development of new PDE5-Is with higher potency, selectivity, and improved pharmacokinetics for enhanced efficacy. This review provides an update on the approved and potential uses, side effects, and published inhibitors, covering binding properties, selectivity, pharmacokinetics, and in vivo efficacy over the past decade, as well as the most recent clinical studies.

## 2. Classification of Phosphodiesterases

PDE superfamily comprises 11 families (PDE1–PDE11) that are encoded by 21 different genes, whose expression are modulated via multiple promotors and messenger RNA (mRNA) alternative splicing generating more than 50 isoforms [1,2]. It is worth noting that PDE12, which cleaves 2′,5′-phosphodiester bond linking adenosines of the 5′-triphosphorylated oligoadenylates, belongs to the C–C chemokine receptor 4 (CCR4)/nocturin family [3] and is not a member of the cyclic nucleotide PDE superfamily. PDE isoforms are classified based on their amino acid sequences, substrate specificities, catalytic and cofactor requirements, kinetic properties, regulatory mechanisms, and tissue distributions [1]. Some PDEs are selective for the hydrolysis of cAMP (PDE 4, 7, and 8) or cGMP (PDE 5, 6 and 9), while others can hydrolyze both cAMP and cGMP (PDE 1, 2, 3, 10 and 11) [4]. PDEs share a conserved catalytic domain (C domain) but differ significantly in their N-terminal regulatory domains. PDEs are mainly regulated via (i) binding of Ca^2+^/calmodulin (PDE1), (ii) phosphorylation/dephosphorylation events (PDE1, 3, 4 and 5) and (iii) allosteric binding of cGMP via GAF domains (PDE2, 5, 6, 10 and 11) [1]. The description of diverse tissue distribution/cell expression and functional significance of PDE isoenzymes is detailed in [5] and is beyond the scope of this review. Notably, such tissue/cellular compartmentalization allows selective PDE inhibitors to exert their effects almost exclusively on the target tissue.

Our focus herein is on the PDE5 family, which is generated by one gene, PDE5A, and has three alternative spliced variants, PDE5A1, 5A2 and 5A3. The three human PDE5 isoforms differ only in the 5’-end of the mRNA and the corresponding N-terminal of the protein. These isoforms have similar phosphorylation sites, allosteric cGMP-binding sites, catalytic domain and cGMP binding and hydrolysis activities [6]. However, PDE5A1 was reported to be more resistant to chemical inhibition than PDE5A2 or PDE5A3. PDE5A1 and PDE5A2 are widely distributed in nearly all tissues, whereas PDE5A3 is confined to vascular smooth muscle cells [2]. 

## 3. Tissues and Organs of High Expression for PDE5

PDE5 is present in virtually all cell types, tissues and organs. PDE5 is highly expressed in the smooth muscle cells of the peripheral arteries and venous vessels and in coronary and pulmonary arteries [2]. In addition, PDE5 is expressed in the vascular smooth muscle cells of the corpora cavernosa of the penis besides spermatozoa, peritubular myoid of Leydig cells and vas deferens in males [7]. PDE5 is widely distributed in the cytoplasmic cell compartment in myometrial cells, endothelial cells and peripheral blood mononuclear cells. It is also expressed in skeletal muscles, cardiomyocytes, platelets, lung, spinal cord, cerebellum, retina, pancreas, prostate, urethra and bladder [1,2,8]. PDE5A1 and PDE5A2 are further expressed in renal vessels, glomeruli, tubular epithelial cells of the renal proximal tubule and medullary collecting duct [9]. Consequently, PDE5 isoforms exhibit diverse and numerous functions both in physiological and pathological conditions. 

## 4. PDE5 Physiological Role

Nitric oxide (NO) is synthesized from the precursor L-arginine through the activities of different NO synthases (neuronal, inducible or endothelial NOS). Intracellularly, NO binds to and activates soluble guanylyl cyclase (sGC), promoting the conversion of guanosine triphosphate (GTP) to the second messenger cyclic guanosine monophosphate (cGMP) [10,11]. Thereafter, cGMP activates protein kinase G (PKG), whose phosphorylation mediates activities of various membrane channels/pumps, leading to decreased calcium influx through L-type calcium channels and increased calcium sequestration, resulting in smooth muscle relaxation and vascular tone modulation [12]. PKG-dependent phosphorylation of other various downstream proteins can regulate further pivotal physiological functions, such as cell differentiation and proliferation, endothelial permeability, ion transport, secretion and gene transcription [13]. 

Given the broad expression and the ability of PDE5 to specifically hydrolyze cGMP, controlling its cellular levels, PDE5 has been proposed as a crucial player in many NO/cGMP/PKG-dependent biological processes such as smooth muscle relaxation, heart muscle contraction, platelet activation/aggregation and immune response [14].

PDE5 inhibition was found to enhance smooth muscle relaxation and vasodilation, which in the penis corpus cavernosum favors erection, in the pulmonary vasculature decreases pulmonary vessels’ pressure, and in the systemic circulation decreases arterial blood pressure [15]. 

In addition, PDE5 is an important regulator of platelet function, whose inhibition increases platelet cGMP levels and augments the ability of NO to inhibit platelet aggregation and activation [16].

Furthermore, PDE5 governs fundamental physiological processes in the kidney. It can regulate renal vascular blood flow by hampering cGMP-mediated vascular relaxation. PDE5 is also a negative regulator for cGMP-dependent natriuresis. Moreover, it increases renin synthesis by degrading cGMP in juxtaglomerular cells [17]. 

## 5. PDE5 as a Drug Target for Disease Treatment

**Figure 1:**
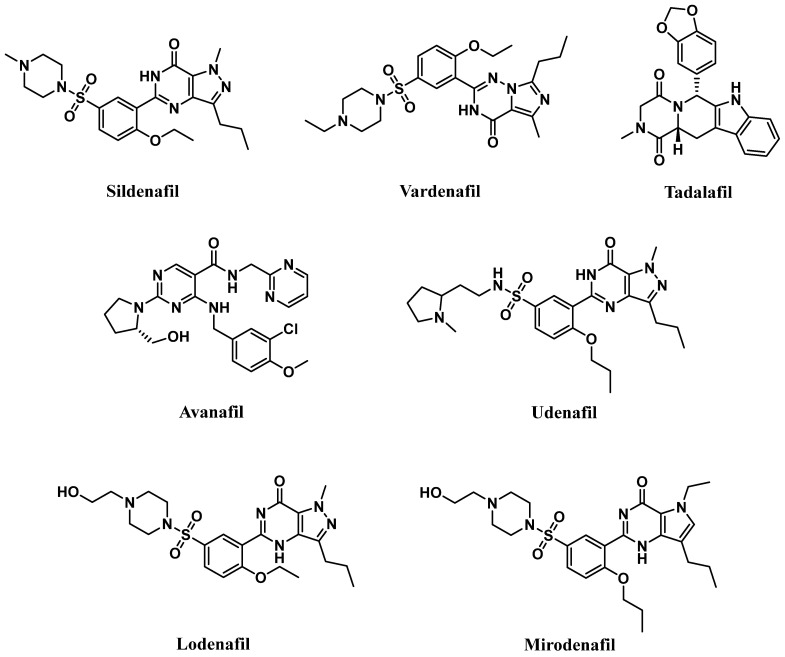
Chemical structures of marketed PDE5 inhibitors.

Competitive PDE5-Is reported so far exclusively bind to the catalytic domain, preventing cGMP hydrolysis and elevating its levels in cells of various tissues [18]. The subsequent activation or restoration of normal NO/cGMP/PKG signaling cascade prompted the use of these inhibitors as therapeutics for several clinical indications. Food and Drug Administration (FDA)-approved PDE5-Is (Figure 1) include (i) sildenafil (approved in 1998 for erectile dysfunction (ED) as Viagra^®^, and in 2005 for pulmonary arterial hypertension (PAH, WHO Group I) as Revatio^®^), (ii) vardenafil (approved in 2003 for ED as Levitra^®^), (iii) tadalafil (approved in 2003 for ED as Cialis^®^, in 2009 for PAH (WHO Group I) as Adcirca^®^ and in 2011 for lower urinary tract symptoms secondary to benign prostatic hyperplasia (LUTS/BPH) with or without ED and the most recent (iv) avanafil (approved in 2012 for ED as Stendra^®^) [19,20]. 

These inhibitors differ in their selectivity, potency, onset and duration of action, cost, administration considerations, precautions, and adverse effects profiles. The relative potency of vardenafil for PDE5 was reported to be the highest (PDE5 IC_50_ of 0.1–0.4 nM), followed by tadalafil (PDE5 IC_50_ of 2 nM), and then sildenafil and avanafil (PDE5 IC_50_ of 4 nM and 4.3–5.2 nM, respectively). They all share a quick onset time in the range of 11–16 min after oral administration, with avanafil advertised as the fastest-acting. Plasma half-lives of sildenafil and vardenafil are similar, about 4 h, and their efficacy of action lasts up to 12 h. Avanafil half-life is shorter, about 3 h, with a maximal duration of action of 6 h. Tadalafil’s half-life is the longest, 17.5 h, with an efficacy maintained for up to 36 h [18,20,21,22]. 

A new generation of PDE5-Is, namely lodenafil, udenafil, and mirodenafil are also available in Brazil and Korea for ED treatment (Figure 1), but none of them have been FDA-approved, yet [23].

Aside from the three FDA-approved clinical indications, PDE5-Is have been intensively investigated for their potential use in the treatment of various emerging indications, such as cancer, central nervous system (CNS) and cardiovascular system (CVS) related diseases, kidney diseases, cystic fibrosis and diabetes, all of which will be discussed herein. 

### 5.1. Approved Clinical Uses of PDE5 Inhibitors

#### 5.1.1. Erectile Dysfunction

**Figure 2:**
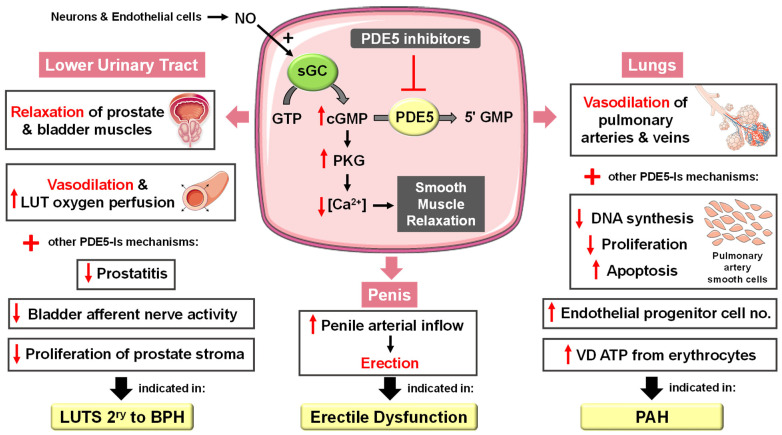
Approved clinical uses of PDE5 inhibitors. Nitric oxide (NO) is produced by neurons and endothelial cells. Inside smooth muscle cells, NO activates soluble guanylyl cyclase (sGC), promoting the conversion of guanosine triphosphate (GTP) to the second messenger cyclic guanosine monophosphate (cGMP). Thereafter, cGMP activates protein kinase G (PKG), whose phosphorylation mediates activities of various membrane channels/pumps, leading to decreased intracellular calcium levels resulting in smooth muscle relaxation (SMR). Phosphodiesterase 5 (PDE5) regulates cGMP levels by degrading it into inactive 5′ guanosine monophosphate (5′ GMP). PDE5-Is can thus enhance the cGMP/PKG pathway, boosting the relaxation of various smooth muscles. In the penis corpus cavernosum, SMR favors erection due to increased penile arterial inflow, and thus PDE5-Is are approved for the treatment of erectile dysfunction. In the lungs, PDE5-Is lead to vasodilation of pulmonary vasculature, which, along with other mechanisms, such as suppressed DNA synthesis and proliferation and enhanced apoptosis of pulmonary artery cells, increased endothelial progenitor cell number, and enhanced release of vasodilating adenosine triphosphate (ATP) from erythrocytes culminate in effectiveness in the treatment of pulmonary arterial hypertension (PAH). In the lower urinary tract (LUT), PDE5-Is mediate prostate and bladder SMR, vasodilation and increased LUT oxygen perfusion. In addition, PDE5-Is could suppress prostatitis, bladder afferent nerve activity and prostate stroma cell proliferation, and thus indicated in the treatment of LUT symptoms secondary to benign prostatic hyperplasia (BPH).

In the corpora cavernosa, parasympathetic stimulation and sexual arousal induce the release of NO from endothelial cells and nitrergic neurons surrounding the arteries and sinusoids, leading to increased cGMP synthesis. PDE5-Is can slow the degradation of penile connective tissue cGMP. This leads to a drop in the intracellular Ca^2+^ levels in the corpus cavernosum smooth muscles, causing their relaxation and a reduction in arterial blood drainage, providing a sufficient degree of penile tumescence and sustaining penile erection (Figure 2). Accordingly, it can be deduced that the action of PDE5-Is requires normal neuronal input into the erectile tissues, as well as unimpaired cavernous endothelial structures [24,25].

PDE5-Is are thus considered the first-line choice for on-demand and chronic treatment of most ED cases. The efficacy and safety of the four FDA-approved PDE5-Is (sildenafil, vardenafil, tadalafil and avanafil) have been confirmed by a multitude of worldwide clinical trials involving thousands of ED patients with diverse etiologies that were documented by several reviews [19,26,27,28,29,30].

It is worth noting that the few differences between sildenafil, tadalafil and vardenafil pharmacokinetics allow tadalafil, with a longer half-life, to be superior in a number of sexual intercourses per pill, while vardenafil and sildenafil exhibited privilege whenever duration of erection, or vascular efficacy and penile hardness are explored [31,32]. 

#### 5.1.2. Pulmonary Arterial Hypertension

PAH is a disease associated with endothelial dysfunction, vascular remodeling and fibrosis that causes gradual progression of pulmonary vascular resistance, ultimately leading to right heart failure. Accordingly, PAH therapies usually aim to enhance vasodilation, suppress cellular hyperproliferation and induce apoptosis [33]. 

PDE5 is highly expressed in the lung vasculature [34]. The fact that lung endothelial NOS is reduced [35] and PDE5 is upregulated in the remodeled pulmonary artery during PAH has proposed PDE5-Is as a potential PAH treatment [36]. A plethora of PDE5 inhibition-mediated mechanisms have been documented (Figure 2) including (i) activation of the NO/cGMP/PKG pathway, resulting in decreased calcium influx through L-type calcium channels and increased calcium sequestration, inducing vasorelaxation [37], (ii) suppression of DNA synthesis and cell proliferation and stimulation of apoptosis of pulmonary artery smooth cells whose proliferation is involved in the pathogenesis of intimal hyperplasia and major vascular lesions in PAH [38], and (iii) increasing circulating endothelial progenitor cell (EPC) number [39]. 

Several clinical studies confirmed the potential of PDE5-Is to improve several hemodynamic and clinical parameters in PAH patients [40,41,42,43], such as diminishing pulmonary artery systolic and mean artery pressure, dyspnea score and gas transfer, pulmonary vascular resistance and cardiac output [44]. Furthermore, PDE5-Is could improve ventilatory efficiency and oxygen uptake kinetics and prevent exercise-induced pulmonary edema [45]. Vardenafil usually exhibits the most rapid effect on pulmonary vasorelaxation, while sildenafil and tadalafil are more selective for pulmonary circulation. Substantial enhancement of arterial oxygenation is mainly observed with sildenafil [46].

Sildenafil, in 2005, and, thereafter, tadalafil have been FDA approved and became first-line therapies for PAH [47], primary or secondary to other connective tissue diseases, such as scleroderma (SSc) or systemic lupus erythematosus (SLE) [34].

PDE5-Is can also be used as combination therapy with other PAH-targeted treatments. The combination of sildenafil and long-term intravenous epoprostenol therapy was superior to epoprostenol monotherapy regarding improved exercise capacity, hemodynamic measurements and prolonged time to clinical worsening [48]. Other combinations, such as tadalafil with the endothelin receptor antagonist ambrisentan and sildenafil with systemic nitrates [49], were proven safe and effective in potentiating vasodilation and reducing mortality in PAH patients. Moreover, combined prostacyclin analogs and PDE5-Is were reported to synergistically enhance the release of the potent vasodilator ATP from PAH erythrocytes [50].

PDE5 inhibition has also emerged as a therapeutic strategy for high-altitude PAH where sildenafil’s ability to reverse hypoxia-mediated pulmonary vasoconstriction was proved to mediate positive results on exercise performance and lung hemodynamics [51,52].

#### 5.1.3. Lower Urinary Tract Symptoms Secondary to Benign Prostatic Hyperplasia 

Several studies have established an association between ED and BPH-related LUTS where alterations in the NO/cGMP pathway, alterations in RhoA/Rho kinase/endothelin signaling, pelvic atherosclerosis, autonomic adrenergic hyperactivity, inflammatory pathways, sex hormones and psychological factors were the major contributing factors [53,54]. Accordingly, attention was drawn towards the development of a single therapy to treat both conditions.

The clinical benefits of chronic PDE5 inhibition on LUTS secondary to BPH, regardless of whether these symptoms are associated with ED, are well documented [55]. These beneficial effects have been correlated to several mechanisms (Figure 2), including (i) stromal smooth muscle relaxation of the prostate and bladder due to modulation of the NO/cGMP pathway in the nitrinergic innervated organs or enhanced generation of relaxing hydrogen sulfide, (ii) significant cGMP-mediated dilatation of local blood vessels, (iii) enhanced LUT oxygen perfusion, (iv) inhibition of afferent nerve activity of bladder, (v) down-regulation of prostate inflammation and (vi) negative regulation of proliferation and trans-differentiation of the prostate stroma [54,56,57].

Many preclinical studies of PDE5 and its inhibitors in the prostate and bladder (reviewed in [58]) could validate the role of PDE5-Is in relaxing prostatic tissue, improving the severity of urinary symptoms, reducing bladder overactivity, decreasing indicators of bladder ischemia, normalizing changes in NOS activity and preventing the accumulation of collagen [59].

Several clinical trials demonstrated that the use of PDE5-Is alone could ameliorate LUTS in the first 12 weeks of treatment, where sildenafil [60], tadalafil [61,62,63] and vardenafil [64] led to a decrease, at different degrees, in the International Prostate Symptom Score (IPPS) scale. In particular, the effects of tadalafil 5 mg once daily versus placebo on LUTS/BPH have been extensively investigated (reviewed by Gacci et al. [65]). Only tadalafil (5 mg once daily) has been licensed for the treatment of LUTS with or without ED.

The combined administration of sildenafil, tadalafil or vardenafil with the α_1_-adrenoceptor antagonists alfuzosin or tamsulosin for the treatment of LUTS/BPH has also been evaluated and was confirmed to often outperform either type of monotherapy [66,67,68,69,70]. Interestingly, a very recent meta-analysis of randomized clinical trials demonstrated that tadalafil could be superior to tamsulosin in treating LUTS/BPH when associated with ED [71].

### 5.2. Emerging and Future Uses of PDE5 Inhibitors

#### 5.2.1. Cancer

**Figure 3:**
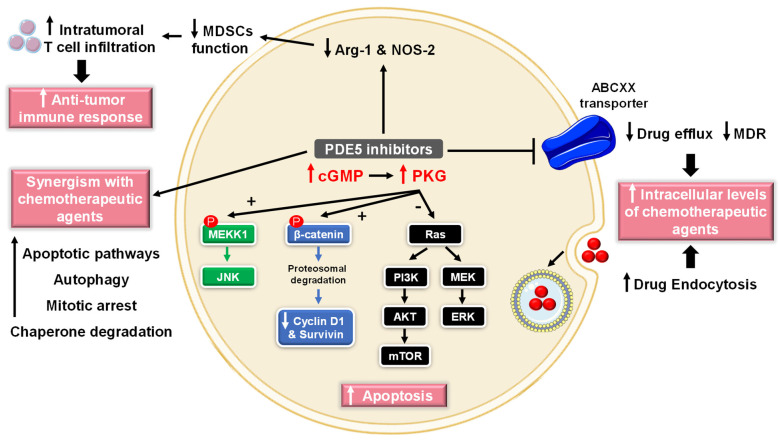
Anti-cancer mechanisms of PDE5 inhibitors. Via activation of the cGMP/PKG signaling cascade, PDE5-Is can induce apoptosis in cancer cells via various pathways; activation of c-Jun NH_2_-terminal kinase (JNK) via phosphorylation of mitogen-activated protein kinase kinase kinase 1 (MEKK1), phosphorylation of β-catenin and inducing its proteosomal degradation which leads to decreased expression of Wnt/β-catenin regulated proteins, such as cyclin D1 and survivin in addition to blocking the phosphoinositide 3-kinase (PI3K)/AKT/mammalian target of rapamycin (mTOR) and the mitogen-activated protein kinase kinase/extracellular signal-regulated kinase (MEK/ERK) signaling pathways. PDE5-Is could also increase intracellular levels of other chemotherapeutic agents via inhibition of the ATP-binding cassette (ABC) transporter-mediated drug efflux, averting multidrug resistance (MDR) in addition to increasing cellular drug uptake via enhancing endocytosis. Moreover, PDE5-Is synergize with other chemotherapeutic agents via boosting various apoptotic, autophagy, mitotic arrest and chaperone degradation pathways. PDE5-Is can also abrogate the function of myeloid-derived suppressor cells (MDSCs) via suppression of arginase-1 (Arg-1) and nitric oxide synthase–2 (NOS-2) production. This results in enhanced intratumoral T-cell infiltration and activation and restores both systemic and tumor-specific immunity. P = phosphorylation.

Numerous studies have reported the role of cGMP in suppressing cell growth and inducing apoptosis and that elevated PDE5 expression is involved in the progression of various tumor types, such as chronic lymphocytic leukemia, colon adenocarcinoma, bladder squamous carcinoma, human papillary thyroid carcinomas, metastatic breast, prostate, pancreatic and lung cancers [72,73,74]. Accordingly, PDE5 has gained attention as a promising target for anticancer drug discovery. Over the last two decades, several pre-clinical and clinical studies revealed potential anti-cancer effects of PDE5-Is [75,76] that were mediated via different mechanisms of action discussed herein (Figure 3).

(1) Cell growth arrest and induction of apoptosis

Sildenafil and vardenafil were reported to induce caspase-dependent apoptosis and antiproliferative effects in B-cell chronic lymphatic leukemia [77]. Moreover, sildenafil was shown to boost intracellular reactive oxygen species (ROS) levels, induce cell cycle arrest, and suppress cell proliferation in colorectal cancer cells [78]. In addition, multiple studies have validated the proapoptotic effects of exisulind (sulindac sulfone) and sulindac sulfide (SS), two metabolites of the non-steroidal anti-inflammatory drug (NSAID) sulindac, in breast, colorectal and metastatic prostate cancers. Exisulind or SS increases the activation of cGMP-dependent PKG, triggering a series of signaling events (Figure 3), including (i) phosphorylation of β-catenin and inducing its proteosomal degradation which leads to decreased expression of Wnt/β-catenin regulated proteins, such as cyclin D1 and survivin, (ii) activation of c-Jun NH_2_-terminal kinase (JNK) via phosphorylation of mitogen-activated protein kinase kinase kinase 1 (MEKK1), and (iii) blocking the phosphoinositide 3-kinase (PI3K)/AKT/mammalian target of rapamycin (mTOR) and the mitogen-activated protein kinase kinase/extracellular signal-regulated kinase (MEK/ERK) signaling pathways, all of which culminate in triggering apoptosis cascade [79,80,81,82,83,84].

(2) Chemotherapy sensitization

Several studies provided evidence that PDE5-Is can increase cellular concentrations of standard chemotherapeutic drugs or even enhance their efficacy within certain tumor cells where a combination of potential agents allows the reduction of dose levels and, consequently, of toxic side effects (Figure 3) [76,85,86,87].

One of the major causes of chemotherapy failure in cancer treatment is multidrug resistance (MDR) attributed to overexpression of the ATP-binding cassette (ABC) transporters, such as P-glycoprotein (ABCB1/P-gp/MDR1), multidrug-resistance proteins (ABCCs/MRPs) and breast cancer resistant protein (ABCG2/BCRP). These transporters actively expel chemotherapeutic agents out of the cancer cell, ameliorating their cellular efficacy [88]. Vardenafil was reported to inhibit the drug efflux in ABCB1-overexpressing cells [89], while sildenafil was effective in opposing the activity of ABCB1 and ABCG2, both attenuating MDR in tumor cells [90].

Another study showed that PDE5-Is can increase cellular uptake of structurally diverse compounds into lung cancer cells both in vitro and in vivo via modulation of endocytosis [85]. Moreover, oral administration of sildenafil and vardenafil was found to actively enhance blood tumor barrier (BTB) permeability and boost the efficacy of chemotherapy in a rat brain tumor model [91]. Vardenafil could also enhance the delivery and therapeutic efficacy of herceptin monoclonal antibodies in mouse models of metastatic HER2/neu-positive brain tumors through stimulating caveolae-mediated endocytosis and micropinocytosis [92].

Besides augmenting the delivery of chemotherapeutic agents, PDE5-Is can suppress tumor growth and induce cell death by synergizing with current chemotherapy medications in treating a wide range of cancers (Figure 3).

Celecoxib and PDE5-Is synergize in a NOS-dependent cyclooxygenase (COX)-independent fashion to kill multiple tumor cell types, including human glioma cells, as well as their associated activated microglia in vitro and could suppress the growth of mammary tumors in vivo. The drug combination increased the levels of autophagy by inactivating mTOR and inducing endoplasmic reticulum (ER) stress responses in these cells [93].

A combination of sildenafil with various standard chemotherapy agents was proved effective in various gastrointestinal/genitourinary cancers, such as bladder and colon cancers [87]. A combination of the topoisomerase inhibitor doxorubicin and sildenafil resulted in increased efficacy against prostate cancer cells through ROS generation and subsequent upregulation of pro-apoptotic proteins Bad and Bax and downregulation of anti-apoptotic proteins Bcl-2 and Bcl-xL, amplifying caspase-mediated apoptotic death [94]. In a later study, sildenafil and vardenafil but not tadalafil were found to induce PDE5-independent apoptotic sensitization to doxorubicin in castration-resistant prostate cancer (CRPC) cells through impairment of both homologous recombination (HR) and non-homologous end joining (NHEJ) DNA repair pathways [95]. Furthermore, both in vitro and in vivo studies suggested that sildenafil could synergistically potentiate vincristine-induced mitotic arrest signaling and sensitize caspase-dependent apoptosis in CRPC cells via a mitochondrial damage pathway [96].

The multi-kinase inhibitors sorafenib/regorafenib in combination with sildenafil were reported to suppress xenograft tumor growth using liver and colon cancer cells in a greater than additive manner via various autophagy and intrinsic and extrinsic apoptotic pathways [97]. In multiple genetically diverse lung cancer cell lines, sildenafil increased the lethality of pemetrexed and sorafenib combination via fully inactivating signaling by multiple cytoprotective proteins, including the AKT/ERK pathways, nuclear factor-κB (NF-κB) and STAT3/STAT5 besides enhancing death receptor expression and activation [98].

Treatment of stem-like glioblastoma cells with a combination of OSU-03012 (a non-COX-2 inhibiting derivative of celecoxib) and sildenafil abolished the expression of multiple oncogenic growth factor receptors and plasma membrane drug efflux pumps and caused rapid degradation of glucose-regulated protein (GRP78) and other chaperones in tumor cells. This downregulates key oncogenic kinases, including PI3K/AKT signaling, leading to tumoricidal effects [99]. Similarly, sildenafil alone or in combination with the heat shock protein 90 (HSP90) inhibitor PU-H71 could alter the expression of HSP90 chaperone followed by degradation of the oncogenic protein kinase D2 impairing proliferation and viability of various tumor cell lines [100]. These studies suggest a combination of PDE5 and chaperone inhibitors as a novel, promising strategy for targeting cancer.

(3) Modulation of antitumor immune response

PDE5 inhibition contrasts tumor-induced immunosuppressive mechanisms and generates a measurable antitumor-immune response that significantly delays tumor progression. Both sildenafil and tadalafil could abrogate the function of myeloid-derived suppressor cells (MDSCs) via suppression of arginase-1 (Arg-1) and nitric oxide synthase–2 (NOS-2) production. This resulted in enhanced intratumoral T-cell infiltration and activation and restored both systemic and tumor-specific immunity in multiple myeloma and head and neck cancer patients (Figure 3) [101,102].

(4) Chemopreventive mechanisms

A nationwide population-based study in Sweden suggested that the use of PDE5-Is was associated with a lower risk of colorectal cancer among male patients with benign colorectal neoplasm [103]. Moreover, two very recent studies provided evidence that sildenafil was more effective than tadalafil in preventing the development and progression of aflatoxin B1-induced hepatocellular carcinoma. This beneficial effect was attributed to a plethora of mechanisms, including (i) improved enzymatic antioxidant system capacity with a concomitant decline in the level of lipid peroxidation, (ii) increase in activity of glutathione S-transferase, (iii) downregulation of glucose transporter 1 (GLUT1) restoring normal declined blood glucose levels in tumor cells, (iv) inhibition of lactate dehydrogenase dependent glycolytic machinery, (v) vasodilation of blood vessels resulting in decreased tumor hypoxia and downregulation of the angiogenesis markers; hypoxia-inducible factor 1-alpha (HIF-1α), transforming growth factor-beta 1 (TGF-β1) and vascular endothelial growth factor A (VEGFA) [104,105]. PDE5-Is have also been shown to suppress the stemness of PC3-derived cancer stem cells (PCSCs) that were confirmed essential for the initiation, progression and recurrence of prostate cancer. cGMP-dependent PKG promotes mammalian sterile 20-like kinase/large tumor suppressor (MST/LATS) kinases, leading to cytosolic degradation of the oncogenic protein Tafazzin (TAZ) and the activation of the Hippo pathway, a crucial player in modulating stemness of PCSCs [106].

#### 5.2.2. CNS Diseases

**Figure 4:**
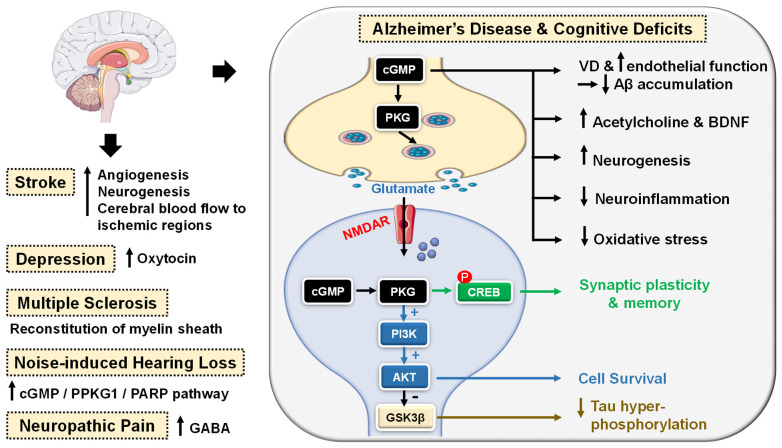
Emerging central nervous system (CNS)-related indications of PDE5 inhibitors. In Alzheimer’s disease (AD) and cognitive deficiency disease models, PDE5 inhibition increases presynaptic cGMP levels, which, through PKG activation, enhances the release of glutamate and activates N-methyl-D-aspartate receptors (NMDAR). On the other hand, postsynaptic PKG activates transcription factor cyclic adenosine monophosphate (cAMP) response element-binding element (CREB), promoting neurotransmission, synaptic plasticity and memory consolidation. PKG also activates the PI3K/AKT signaling pathway that mediates neuroprotection via the inhibition of apoptosis and also suppresses tau hyper-phosphorylation via inhibition of glycogen synthase kinase-3 beta (GSK3β). Elevated cGMP levels exhibit other cognitive enhancement mechanisms, such as vasodilation, which improves or maintains cerebrovascular endothelial function, preventing Aβ amyloid accumulation, rise in acetylcholine (ACh) and brain-derived neurotrophic factor (BDNF) levels in the cortex, striatum, and other areas of the brain, facilitation of neurogenesis, suppression of neuroinflammation and oxidative stress, all averting neuronal loss. In strokes, PDE5-Is could induce angiogenesis and neurogenesis and enhance cerebral blood flow to ischemic regions. PDE5-Is have anxiolytic effects in part due to enhanced oxytocin release. Moreover, PDE5-Is can promote efficient reconstitution of the myelin sheath and govern the Inflammatory processes involved in demyelination models of multiple sclerosis. PDE5-Is are also beneficial in noise-induced hearing loss via activating cGMP/protein kinase cGMP-dependent 1/poly (ADP-ribose) polymerase (cGMP/PRKG1/PARP) signaling in response to traumas in cochlea sensory cells. PDE5-Is exhibit pain-relieving effects in neuropathic pain models via enhanced release of gamma-aminobutyric acid (GABA). P = phosphorylation.

cGMP/PKG signaling has been regarded as a central mechanism of neuroinflammation, neurodegeneration and cognitive disorders [106,107]. Accordingly, PDE5-Is have gained growing attention as potential therapeutic agents for the treatment of several CNS-related diseases, such as Alzheimer’s disease (AD), cognitive deficits, strokes, multiple sclerosis (MS), depression, noise-induced hearing loss (NIHL) and neuropathic pain that will all be discussed in this section (Figure 4). 

PDE5 inhibition increases presynaptic cGMP levels, which, through PKG activation, enhances the release of glutamate and activates N-methyl-D-aspartate (NMDA) receptors. On the other hand, postsynaptic PKG activates transcription factor cyclic adenosine monophosphate (cAMP) response element-binding element (CREB), promoting neurotransmission, synaptic plasticity and memory consolidation [108,109]. PKG also activates the PI3K/AKT signaling pathway that mediates neuroprotection via the inhibition of apoptosis (Figure 4) [110].

The upregulation of PDE5 expression in the brains of AD patients and the subsequent drop in cGMP levels have been linked to the elevation of Aβ amyloid peptide, whose deposition in the brain is the main hallmark of AD [111]. Sabayan et al. described PDE5-Is as disease-modifying agents against AD and proposed three main mechanisms for their action: (i) vasodilation, which improves or maintains cerebrovascular endothelial function preventing Aβ amyloid accumulation; (ii) cGMP-dependent rise in acetylcholine (ACh) levels in the cortex, striatum, and other areas of the brain, reversing low-ACh associated memory and cognitive deficits in AD, and finally (iii) inhibition of apoptosis and facilitation of neurogenesis averting neuronal loss (Figure 4) [112].

For example, chronic administration of sildenafil completely reversed cognitive impairment in Tg2576 transgenic mice without changing Aβ load. The underlying mechanism involved suppression of tau hyperphosphorylation and inhibition of glycogen synthase kinase 3β (GSK3β) and cyclin-dependent kinase 5 (CDK5) [113]. In addition, Puzzo et al. and Zhang et al. showed that chronic administration of sildenafil in amyloid precursor protein/presenilin-1 (APP/PS1) transgenic mice could reverse AD-related cognitive deficits and synaptic dysfunction via improving cGMP/PKG/CREB signaling, inhibiting neuroinflammation and reducing hippocampal Aβ levels [114,115].

Chronic treatment with tadalafil even exhibited a higher beneficial effect, probably due to its longer half-life and could improve spatial memory in the J20 mouse model of AD by decreasing tau protein via the activation of the AKT/GSK3β pathway [116]. Most recently, mirodenafil was reported to ameliorate Aβ-induced AD pathology and improve cognitive behavior in the APP-C105 mouse model through the modulation of the cGMP/PKG/CREB signaling pathway, GSK-3β activity, glucocorticoid receptor transcriptional activity and Wnt/β-catenin signaling in neuronal cells (Figure 4) [107].

Preclinical studies proved that PDE5-Is could boost memory and synaptic plasticity by augmenting the NO/cGMP/PKG pathway [107,117]. In mouse models with induced cognitive deficits, sildenafil could improve novel object recognition, ameliorate cognitive impairment and upregulate the brain-derived neurotrophic factor (BDNF), contributing to neuroprotective effects [118,119]. Another study showed the potential of sildenafil to defy neurological stress, increase neuroprotection and restore cognitive functions in the hippocampus region of noise alone-induced mice via modulation of cGMP/PKG/CREB and p25/CDK5 pathways and induction of various free radical scavengers in the brain of stressed mice [120]. A similar alleviation n of oxidative stress in the hippocampus of aged mice has been observed upon chronic tadalafil administration as well (Figure 4) [121].

Very recent reviews by Liu et al. [122] and Zuccarello et al. [123] summarized clinical trials of PDE5-Is in cognition and AD. However, none of the investigated drugs has reached the market for those indications so far.

Numerous animal models investigated the potential role of PDE5 inhibition in stroke. In these studies, PDE5-Is could induce angiogenesis, enhance cerebral blood flow to the ischemic region, increase neurogenesis and advanced functional post-stroke recovery [124,125,126]. In particular, sildenafil treatment for two weeks (25 mg daily) was proven safe in patients who suffered mild to moderate strokes [127]. Additionally, tadalafil could attenuate ischemia-induced short-term memory impairment by suppressing ischemia-induced neuronal apoptosis [128]. 

Further mechanisms for PDE5 inhibition-induced neurogenesis have been reported and include AKT/GSK3β phosphorylation [129] or triggering proliferation of neural stem cells (NSC) via a mitogen-activated protein kinase (MAPK) dependent signaling cascade [130]. 

Moreover, preclinical studies have provided further evidence of sildenafil’s neuroprotective potential observed against Aβ amyloid-induced mitochondrial toxicity [131]. Additionally, 3-nitropropionic acid-induced behavioral and biochemical toxicities in a Huntington’s disease rat model [132].

Interestingly, a clinical study showed that single-dose sildenafil could improve regional cerebrovascular reactivity deficits in chronic traumatic brain injury patients as well [133].

Sildenafil has also been reported to promote efficient reconstitution of the myelin sheath and govern the inflammatory processes involved in demyelination models of MS [134]. Sildenafil could also normalize experimental autoimmune encephalomyelitis in MS mouse models [135].

Administration of sildenafil or tadalafil could yield significant anxiolytic-like effects in rodent genetic models of depression as well due to chronic activation of the NO/cGMP system [136,137]. Another reported mechanism for the antidepressant-like effect of sildenafil involved the activation of the oxytocin [138].

Jaumann et al. unveiled a potential protective role of activated cGMP/protein kinase cGMP-dependent 1/poly (ADP-ribose) polymerase (cGMP/PRKG1/PARP) signaling in response to traumas in cochlea sensory cells of various animal models. These data suggested PDE5 as a valid target for the improvement of NIHL. In particular, treatment of rodent models with vardenafil before or 6 h after acoustic trauma was shown to diminish auditory-evoked brain stream response thresholds in all frequency ranges tested [139].

Several animal studies have also proposed a beneficial pain-relieving effect of PDE5-Is in models of lesional [140,141] or metabolic neuropathic pain [142]. Sildenafil could ameliorate neuropathic pain symptoms in patients with diabetic peripheral neuropathy [143] and showed an antinociceptive effect in Sprague–Dawley male rats’ neuropathic pain models [144]. Mechanistically, this analgesic effect has been correlated to cGMP-dependent enhanced release of gamma-aminobutyric acid (GABA) [144].

#### 5.2.3. Cardiovascular Diseases

Cardiomyocytes normally express a minimal basal level of PDE5. However, cardiac PDE5 expression was reported to be upregulated in hypertrophic, dilated, and ischemic cardiomyopathy and in congestive heart failure [47,145,146]. The protective effects of PDE5-Is against myocardial infarction (MI), cardiac ischemic and reperfusion (I/R) injury were validated in many in vitro studies with sildenafil [147], tadalafil [148,149], and vardenafil [150]. When given either prior to occlusion or at reperfusion, these PDE5-Is could reduce infarct size, attenuate cardiac hypertrophy, improve left ventricular (LV) function and prevent progression to heart failure. 

In a mouse model, sildenafil exhibited a preconditioning effect to protect the heart against necrosis and apoptosis [151]. Another study suggested that the cardioprotective effect of sildenafil in female mice is estrogen-dependent as ovariectomy suppressed its anti-hypertrophic effect [152].

Intramyocardial transplantation of human adipose stem cells (ASCs) is regarded as a potential treatment for post-ischemic heart failure. Hoke et al. showed that preconditioning of ASCs with sildenafil could trigger the release of significantly high levels of pro-angiogenic or pro-survival growth factors, which enhance ASCs survival and therapeutic efficacy in cardiac ischemic microenvironment, allowing successful cardiac regeneration [153].

Tadalafil also showed cardioprotective effects via PKG-dependent generation of hydrogen sulfide [154]. Moreover, tadalafil was suggested to be clinically beneficial in metabolic syndrome (MetS) patients who are at high risk for CVS diseases where it improved insulin sensitivity, lowered circulating lipids, improved LV diastolic dysfunction and protected against I/R injury in MetS mice [155].

PDE5-Is manifested more significant protective effects against advanced heart failure (HF) with reduced ejection fraction than in HF with preserved ejection fraction [156]. Sildenafil could suppress chamber and myocyte hypertrophy and reverse preestablished hypertrophy in mice exposed to chronic pressure overload. This anti-hypertrophic effect was mediated by the deactivation of multiple signaling pathways, including the calcineurin/nuclear factor of activated T-cells (NFAT), PI3K/AKT, and ERK1/2 signaling pathways [157]. Furthermore, several clinical studies have confirmed the potential role of sildenafil in improving cardiac output, endothelial function, muscle perfusion, and exercise ventilatory and aerobic efficiencies in systolic HF patients [158,159,160].

Moreover, prophylactic treatment with either sildenafil or tadalafil improved cardiac contractile function and survival by attenuating doxorubicin-induced apoptosis and cardiac oxidative stress without interfering with the antitumor efficacy of doxorubicin in both in vitro and in vivo tumor models [161,162].

PDE5 inhibition could govern two crucial vascular manifestations of essential hypertension as well via diminishing blood pressure and improving arterial stiffness and endothelial dysfunction [163].

In addition, sildenafil elicited a significant decrease in inducible ventricular tachycardia and ventricular fibrillation in animal models and demonstrated protection against ventricular arrhythmias associated with the early stages of cardiac ischemia or following MI [164,165].

PDE5-Is could also inhibit platelet activation and aggregation [166,167]. Sildenafil, in particular, was demonstrated to (i) improve coronary patency in an animal model [168], (ii) reduce thrombosis, thromboembolic events, and the risk of thrombotic strokes in a clinical study [169], and (iii) potentiate the anti-aggregation effect of NO donors via cGMP-dependent and independent pathways [170].

Owing to their vasoactive effects, both sildenafil and tadalafil showed advantages in minimizing skin flap necrosis and in preventing extremity and flap ischemia in patients with Raynaud’s phenomenon and with scleroderma [171,172].

Kloner et al. thoroughly investigated the cardiovascular safety profile of PDE5-Is published in the last two decades and confirmed their safety [173].

**Figure 5:**
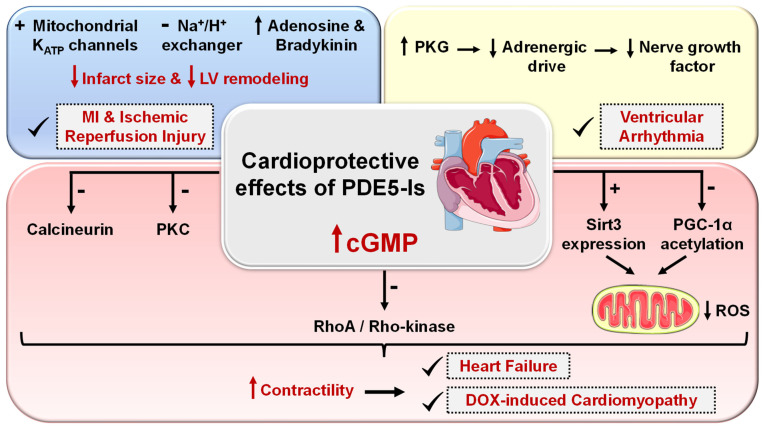
Cardioprotective effects of PDE5 inhibitors. PDE5-Is restore high cGMP levels in cardiomyocytes that govern diverse downstream cardioprotective mechanisms: (i) PKG-dependent opening of mitochondrial and sarcolemmal ATP-sensitive potassium channels, inhibition of Na^+^/H^+^-exchanger and release of endogenous cardioprotective molecules, such as adenosine, bradykinin from endothelial cells; resulting in reduced infarct size and hampered post-infarct left ventricular (LV) remodeling. All are beneficial for ischemic post-conditioning protection against myocardial infarction (MI) and ischemic reperfusion (I/R) injury, (ii) PKG-dependent suppression of adrenergic drive which reduces nerve growth factor leading to anti-arrhythmic effects, (iii) suppression of protein kinase C (PKC), calcineurin and RhoA/Rho-kinase pathways and (vi) suppression of oxidative stress and improving mitochondrial ultrastructure and function via increased sirtuin-3 (Sirt3) protein expression and decreased peroxisome proliferator-activated receptor gamma coactivator 1-alpha (PGC-1α) acetylation, all culminating in improved cardiac contractility and protection against heart failure (HF) and doxorubicin(dox)-induced cardiomyopathy.

Cardio protection achieved by PDE5-Is is mainly attributed to restoring high cGMP levels in cardiomyocytes that govern diverse cardioprotective mechanisms as follows (Figure 5): (i) vascular tone regulation and release of endogenous cardioprotective molecules, such as adenosine, bradykinin and phenylephrine from endothelial cells [174], (ii) PKG-dependent opening of mitochondrial and sarcolemmal ATP-sensitive potassium channels modulating calcium homeostasis and survival of cardiomyocytes, preventing post-infarct LV remodeling and reducing infarct size [175,176], (iii) PKG-dependent suppression of adrenergic drive which reduces nerve growth factor leading to anti-arrhythmic effects [164], (iv) ischemic post-conditioning protection against MI via PKG-dependent enhancement of Na^+^/K^+^-ATPase activity [177] and inhibition of Na^+^/H^+^-exchanger, delaying normalization of pH during reperfusion [178], (v) suppression of protein kinase C (PKC) and calcineurin culminating in improved contractility and protection against HF [179], (vi) improving mitochondrial ultrastructure and function via increased sirtuin-3 (Sirt3) protein expression and decreased peroxisome proliferator-activated receptor gamma coactivator 1-alpha (PGC-1α) acetylation protecting against post-infarction HF [180], and (vii) inhibition of RhoA/Rho-kinase pathway [181].

#### 5.2.4. Kidney Diseases

Coskuner and coauthor [17] and Afsar et al. [182] thoroughly investigated the renoprotective benefits of PDE5-Is in kidney-related clinical conditions, such as diabetic or nephrotoxic nephropathy, renal ischemia/reperfusion injury, renovascular hypertension and chronic kidney disease. Most reported preclinical studies highlighted a promising potential of PDE5-Is to improve renal function and histopathological changes via collaborative mechanisms, including antioxidative, anti-inflammatory, anti-apoptotic, antifibrotic pathways along with suppression of DNA damage and improving renal blood flow, NOS levels, endothelial function and mitochondrial biogenesis. Most recently, tadalafil was also reported to avert the onset of ureter inflammation and urothelial degeneration in a unilateral ureteral obstruction animal model via modulation of various histopathologic and biochemical changes [183].

#### 5.2.5. Cystic Fibrosis

Cystic fibrosis (CF) is a disease that is caused by a mutation in the CF transmembrane conductance regulator (CFTR) gene “F508del allele” that encodes the main chloride channel expressed in epithelia, which leads to a reduced transepithelial chloride transport in multiple organs, such as pancreas, intestine, kidney, liver and most significantly lungs. This results in abnormal mucociliary clearance and endosomal hyper-acidification along with obstruction, infection and excessive proinflammatory responses that progressively damage the respective organ function and structure [184]. 

Several preclinical and clinical studies highlighted that PDE5-Is can correct the majority of the known pathological defects in CF, where tadalafil showed the highest efficacy, while vardenafil granted prolonged effects after a single therapeutic dose [185,186]. The efficacy of PDE5-Is in CF could be correlated to one or more of the following mechanisms: (i) correction of the mislocalization of the mutant CFTR protein, restoring normal transepithelial chloride transport [187,188,189], (ii) normalizing the excessive proinflammatory responses via downregulation of M1 markers, tumor necrosis factor (TNF)-*α* and inducible NOS-2 [190,191], (iii) reversing endosomal hyper-acidification via elevating cGMP levels [192], (iv) improving endothelial function via promoting NOS-3 phosphorylation in endothelial cells [193], and (v) reducing adhesion of bacterial pathogens to respiratory epithelial cells [190].

#### 5.2.6. Diabetes

Das et al. have summarized the potential protective roles of PDE5-Is against several diabetes-related pathologies including (i) prevention of diabetic neuropathy and vasculopathy via improving endothelial function, (ii) protection against I/R injury in diabetic heart via an AMP-activated protein kinase/Sirt1/PGC-1α (AMPK/Sirt1/PGC-1α) cytoprotective signaling cascade, along with (iii) antioxidant and anti-inflammatory effects in diabetic hearts [86].

A meta-analysis of randomized controlled trials has also validated PDE5-Is as effective and safe medications for the treatment of sexual dysfunction in patients with diabetes mellitus suffering from ED [194].

Most recently, a combination of tadalafil and hydrochloroquine successfully improved several Type 2 diabetes-related clinical parameters, including a drop in fasting blood glucose and lipid levels, a rise in plasma insulin and insulin-like growth factor-1 levels and improved insulin sensitivity. Interestingly, pretreatment with the same combination showed a potential to diminish the rate and severity of COVID-19 infection in vulnerable diabetic patients [195].

#### 5.2.7. Miscellaneous Indications

Several studies have demonstrated the efficacy of the combined administration of sildenafil with selective serotonin reuptake inhibitors (SSRIs), such as paroxetine and sertraline, for the treatment of premature ejaculation [196]. Moreover, PDE5-Is prompted penile rigidity and recovery of erections in the post-ejaculatory period [197]. Details of related preclinical and clinical trials were further elaborated by the reviews [23,198].

Long-term chronic administration of PDE5-Is could also avert the progression of fibrotic plaques and halt corporal fibrosis in animal models of Peyronie’s disease [199,200]. 

In addition, prolonged administration of low-dose PDE5-Is exhibited a promising beneficial effect in the treatment of male infertility. Sildenafil and vardenafil, in particular, could enhance Leydig cells’ secretory and steroidogenic functions, augmenting sperm concentration and the percentages of motile and morphologically normal sperm [201,202,203]. An increase in serum testosterone levels by both inhibitors has been reported as well [204].

**Figure 6:**
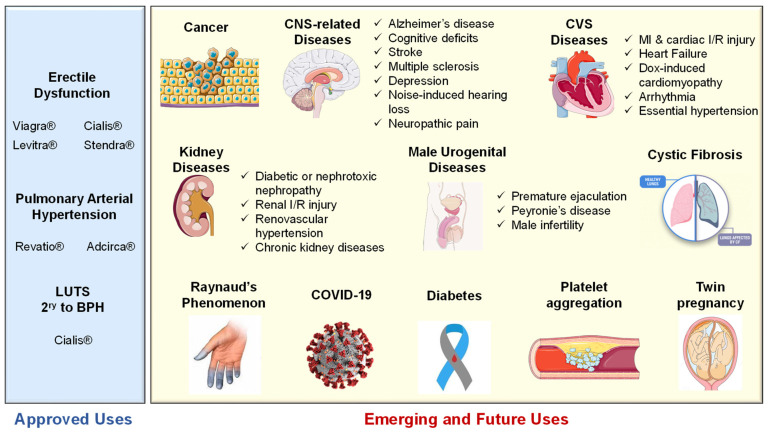
Summary of approved and emerging/future uses of PDE5 inhibitors.

Interestingly, tadalafil was proven safe to improve selective fetal growth restriction, a condition of twin pregnancy in which the development of one fetus is restricted, without severe side effects in the mothers or neonates [205]. Most recently, Isidori et al. collaborated evidence possibly linking the NO/cGMP/PDE5 axis to the pathophysiology of coronavirus disease (COVID-19) and suggested the repurposing of PDE5-Is as a treatment strategy to halt the progression of COVID-19 via diverse immunomodulatory mechanisms [206]. All reported FDA-approved and emerging uses of PDE5-Is are summarized in Figure 6.

### 5.3. Side Effects and Contraindications of PDE5 Inhibitors 

The use of PDE5-Is is usually associated with some common side effects, which include headache, flushing, dyspepsia, visual disturbances, back pain, myalgia, tachycardia, and nasal congestion [207]. Most of these side effects are due to the inhibition of PDEs other than PDE5, visual disturbances are associated with PDE6 inhibition and back pain and myalgia are attributed to the inhibition of PDE11. Nevertheless, these side effects rarely led to discontinuation of the treatment. 

Other less known, seldom encountered serious side effects have been reported concomitant to the use of PDE5-Is are highlighted in the following lines.

(i) Although PDE5 is reported as a promising target for anti-cancer therapy, as explained earlier, the prolonged use of PDE5-Is has been linked to an increased risk of melanoma. Lie and co-workers reported an association between sildenafil use and an increased risk of melanoma in a prospective cohort study conducted on 25,848 men [208]. Several other cohorts and case-control studies have also reported a correlation between the use of sildenafil and tadalafil and the increased risk of melanoma [209,210]. However, this association between the prolonged use of PDE5-Is and the development of cancer was only reported for melanoma; even the risk of other types of skin cancer, such as squamous cell carcinoma and basal cell carcinoma, was not correlated to the use of PDE5-Is [211].
(ii) Visual disturbances have been usually reported with the use of PDE5-Is because of PDE6 inhibition. However, several studies have reported more serious ophthalmologic side effects associated with the use of PDE5-Is, which include non-arteritic anterior ischemic optic neuropathy (NAION), which may eventually lead to vision loss [212]. Two case-crossover studies have shown a two-fold increase in the risk of NAION in men using PDE5-Is, and currently, all PDE5Is (Viagra®, Cialis®, Levitra® and Spedra®) mention NAION as a caution in their summary of product characteristics [213,214].
(iii) Moreover, sensorineural hearing loss (SSHL) has been associated with the prolonged use of PDE5-Is. Two in vivo studies have shown that the prolonged use of sildenafil could lead to hearing loss in mice and rats [215,216]; in addition, published trials and pharmacovigilance agencies reported 47 cases of SSHL as a result of prolonged administration of sildenafil [217], and more specifically, Maddox et al. reported two cases of SSHL due to daily use of tadalafil 10 mg and sildenafil 50 mg + tadalafil 10 mg use where both patients did not recover after a follow-up [218]. Both NAION and SSHL are of unknown pathophysiology.
(iv) Priapism (prolonged erection of the penis) is another less common side effect reported with the prolonged use of PDE5-Is, as only a few cases have been reported for priapism associated with the use of PDE5-Is [219]. The risk of priapism increases in the case of concomitant use of other ED medications along with the PDE5-Is.

Not only can these side effects potentially restrict the utilization of PDE5-Is, but PDE5-Is are also contradicted in the presence of various cardiovascular disorders. Given that approximately one out of every thirteen individuals is estimated to have a cardiovascular disorder, and considering that there are around 620 million people globally living with cardiovascular conditions, it becomes evident that this is a significant concern. Clinical guidelines dictate that the use of PDE5-Is is not recommended in cases of advanced congestive heart failure, unstable or treatment-resistant angina pectoris, recent myocardial infarction, high-risk arrhythmias, obstructive hypertrophic cardiomyopathy, and severe valve diseases, particularly aortic stenosis [220].

## 6. PDE5 Inhibitors

The PDE5 enzyme is a homodimer that includes three main sites [221]: (i) an allosteric site, which consists of two regulatory GAF domains (GAF-A and GAF-B), both are regarded as allosteric binding regions for cGMP, (ii) a phosphorylation site (at Ser92) which plays a role in enzyme activation, and (iii) a catalytic site, which is located at the C-terminal end of the protein (amino acid residues: 535–860) and contains the divalent metal (Zn^2+^ and possibly, Mg^2+^) and the active site of PDE5. 

The catalytic site of PDE11 is the most similar one to that of PDE5 among all other PDEs, while PDE6 shares a similar amino acid sequence and a secondary structure of the catalytic site to PDE5; therefore, PDE6 and PDE11 are the two most common off-targets for PDE5-Is [221]. PDE6 is a key effector enzyme for the phototransduction cascade in the rod and cone segments of the retina in the mammalian eyes. It has a function in visual transduction and response to light [221]. As for PDE11, not much information is available about its physiological functions. However, it is reported to be localized in skeletal muscles, prostate, and the testes [222].

**Figure 7:**
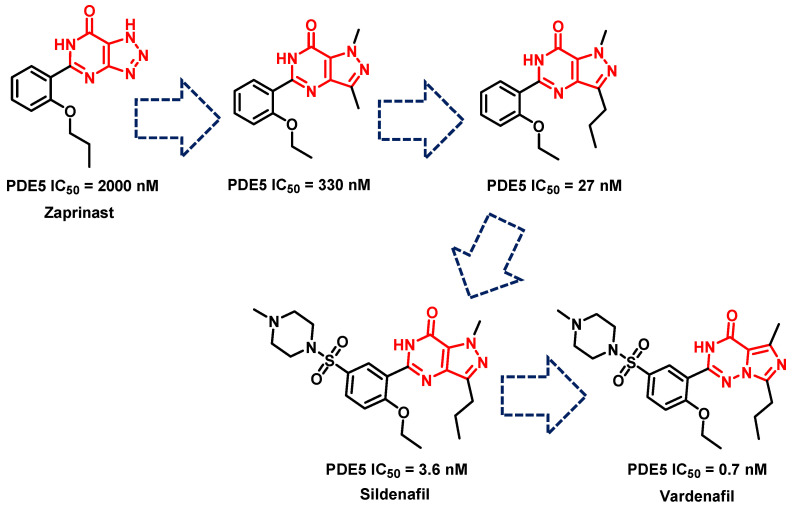
The discovery of sildenafil and vardenafil.

Prior to 2012, the three most important PDE5-Is were sildenafil, vardenafil and tadalafil. Sildenafil was discovered based on the optimization of zaprinast (an anti-allergic drug), which was one of the first PDE5-Is to be reported in the literature; however, it showed only moderate activity against PDE5 (IC_50_ = 2000 nM), as well as low selectivity for PDE5 over PDE1 (SI = 4.7). Several rounds of optimization led to the discovery of sildenafil with a much-improved potency over PDE5 (IC_50_ = 3.6 nM), higher selectivity for PDE5 over PDE1 than zaprinast (SI = 72.2), and improved solubility and in vivo characteristics (Figure 7) [223]. Isosteric replacement of the pyrazolopyrimidinone core with an imidazotriazinone scaffold led to the discovery of vardenafil; vardenafil was more potent (IC_50_ = 0.7 nM) and selective (SI = 257) for PDE5 over PDE1 than sildenafil (Figure 7) [224]. Both sildenafil and vardenafil were reported to prompt visual disorders, such as functional blindness, blue (cynopsia), blurred vision and enhanced light sensitivity, all attributed to the cross-reactivity with the PDE6 catalytic site [225,226]. 

**Figure 8:**
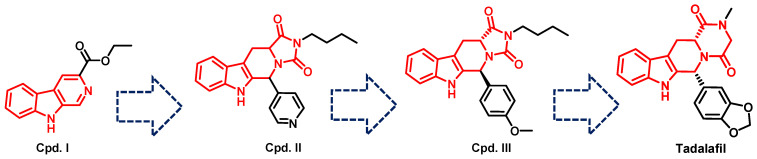
The discovery of tadalafil.

Tadalafil’s development was based on the β-carboline scaffold. The discovery of tadalafil started from the ethyl β-carboline-3-carboxylate (I) that displayed a moderate PDE5 inhibitory activity (IC_50_ = 800 nM) [227]. Reduction of the β-carboline scaffold of cpd. I to a tetahydrabetacarboline (THβC) and extending the structure with a hydantoin ring (cpd. II) improved the PDE5 inhibitory activity (IC_50_ = 300 nM), Figure 8 [227]. Adopting various substituted phenyls at position 6 of the tetrahydro-β-carboline scaffold of cpd. II led to the discovery of a highly potent and selective PDE5 inhibitor (cpd. III, Figure 8) with an IC_50_ of 5 nM and a selectivity index of more than 2000 for PDE5 over PDEs 1–4, with the ability to increase intracellular cGMP levels in rat smooth muscle cells (EC_50_ = 1 µM). However, cpd. III displayed poor hypotensive activity in spontaneous hypertensive rats model after oral administration (30 mg/kg), indicating its poor oral absorption [227]. The modification of the hydantoin ring of cpd. III to a piperazinedione, as well as incorporating the 1,3-benzodioxole moiety at position 6 of the THβC scaffold led to the discovery of tadalafil which displayed higher cellular activity in rat smooth muscle cells (increased intracellular cGMP with an EC_50_ of 0.15 µM), and long-lasting blood pressure lowering activity in the spontaneously hypertensive rat model lasting for 7 h after an oral dose of 5 mg/kg [228].

Due to these adverse effects reported for sildenafil, vardenafil and tadalafil that are related mainly to their cross-reactivity with other PDEs, great attention has grown recently towards the design of more selective PDE5-Is. 

In the coming sections, we discuss PDE5-Is reported since 2012, including PDE5-Is with dual pharmacological activities and those developed for radiodiagnosis. 

### 6.1. PDE5 Inhibitors

#### 6.1.1. Pyrimidinones

Wang et al. applied a structure simplification strategy to sildenafil to produce several pyrimidine-4(3*H*)-one derivative as PDE5-Is with a general scaffold (**1**). Hydrophobic groups were a much more preferred substitution at R^2^ than hydrophilic ones, with ethyl and isopropyl being the best substituents. The introduction of a small aliphatic group or a halogen atom except fluorine at R^1^ led to a huge boost in PDE5 inhibitory activity. At R^3,^ an n-propyl group was optimum for activity. Compound **2** was the most potent PDE5 inhibitor of the series with an IC_50_ of 1.6 nM (2.5 times more potent than sildenafil) [229].

Compound **2** was further tested against 11 PDE isoforms to evaluate its selectivity. It showed no significant inhibition against PDE2, PDE3, PDE4, PDE7A1, PDE8A1, PDE9A2, and PDE10A2 at 10 μM, and selectivity factors of 2127, 469 and 29 for PDE5 over PDE11A4, PDE1 and PDE6C, respectively. In comparison to sildenafil, compound **2** showed a slightly better selectivity profile.

Compound **2** was further evaluated in vivo. Despite having a low oral bioavailability (23%), which is 10% lower than that of sildenafil, it showed good efficacy in a rat model of erection. After 30 min of an oral administration of a dose of 10 mg/kg, both intracavernous pressure and arterial blood pressure were significantly elevated in the rat model [229].

To improve the selectivity of this class of compounds, specifically over PDE6, Gong et al. focused on structural modifications involving the sulfamide part of the molecule. Despite being equipotent to compound **2**, compound **3** (IC_50_ = 1.7 nM) was far more selective for PDE5 over PDE6 with a selectivity factor of 941. However, the in vivo efficacy of compound **3** was not evaluated [230]. Moreover, Gong et al. explored the different binding modes of the pyrimidinone scaffold through cocrystal structures of cpds. **4** (PDB: 4I9Z) and **5** (PDB: 4IA0) with the PDE5 active site [230]. See the crystal structures/docking section.


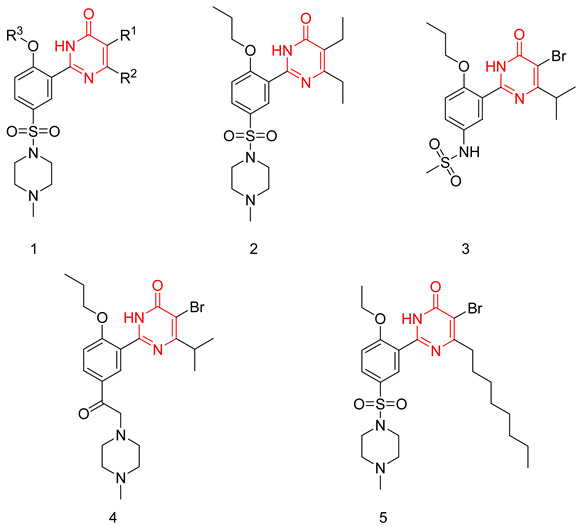


#### 6.1.2. Aminopyrimidines

The aminopyrimidine scaffold was extensively explored by Sakamoto et al., aiming to produce potent and selective PDE5-Is [231,232]. The synthesized derivatives had a general structure (**6**), where their potency was tested on a PDE5 enzyme isolated from a canine lung, their selectivity was evaluated through testing on a light-activated bovine retina PDE6, and their in vivo efficacy was evaluated through testing their ability to induce relaxant effects on an isolated rabbit corpus cavernosum [231,232]. 

The first series of compounds developed by Sakamoto et al. were 5-(3,4,5-trimethoxybenzoyl)-4-aminopyrimidine derivatives. T-6932 (**7**) was the standout compound of the series with a PDE5 IC_50_ of 0.13 nM and a selectivity factor of 2400 over PDE6; however, compound **7** had a moderate in vivo efficacy with an EC_30_ of 53 nM, which was explained by its high clogP value (4.58). On the contrary, compound **8** was the most effective in vivo with an EC_30_ of 3.1 nM (clogP = 3.63). In comparison to sildenafil (EC_30_ = 8.7 nM), it is three times more effective in vivo. However, it was 26 times less potent and four times less selective than **7**. 


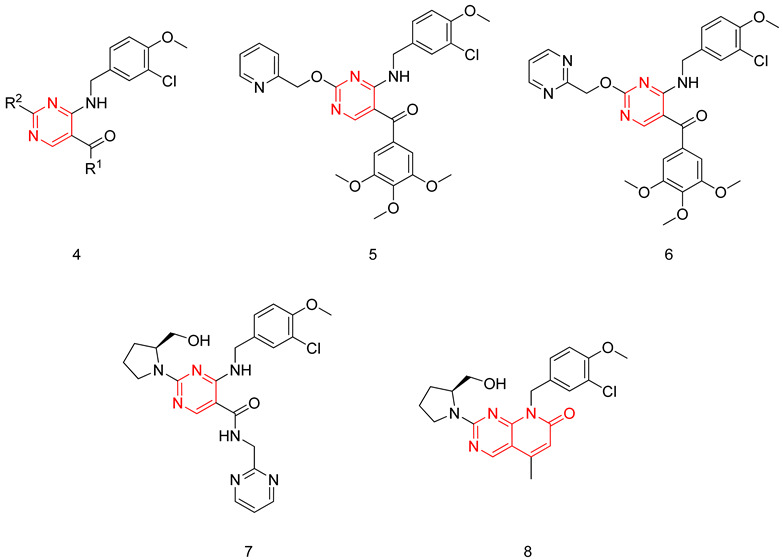


In the next stage of compound development, Sakamoto et al. focused on improving the in vivo efficacy of T-6932 by reducing its lipophilicity. This was done by replacing the 3,4,5-trimethoxyphenyl moiety at R^1^ with several heteroarylmethylamino and hydroxylamine groups, as it was proven in the same study that the 3,4,5-trimethoxyphenyl at R^1^ is not crucial for PDE5 inhibitory activity. Incorporation of these substituents together with a 2-pyridylmethyloxy group at R^2^ maintained the potency against PDE5 but had a negative impact on the selectivity. This was overcome by the introduction of several secondary amines having hydroxyl groups at R^2^. The incorporation of an (*S*)-2-hydroxymethyl-pyrrolidin-1-yl group at R^2^ led to the discovery of avanafil (**9**) with an IC_50_ of 5.2 nM and a selectivity factor of 4000 for PDE5 over PDE6. Avanafil had a much more improved clogP value (2.36) in comparison to T-6932, which could explain its remarkable in vivo efficacy (EC_30_ = 2.1 nM) [232]. 

Avanafil was further tested on the other PDE isoforms, where it showed an excellent selectivity profile, with a selectivity factor of 121 for PDE5 over trypsin-activated PDE6, which is higher than that of sildenafil (16) and vardenafil (21) but lower than that of tadalafil (550). However, avanafil holds the advantage over tadalafil with respect to the selectivity over PDE11 as it has a selectivity factor of more than 19,231, while tadalafil has a selectivity factor of only 25. Moreover, avanafil showed a selectivity factor of more than 1000 over all other PDE isoforms [232].

Avanafil also demonstrated an excellent pharmacokinetic profile, where it possessed a faster onset of action than sildenafil, as well as a short duration of action, improving the tolerability of the drug [232]. The high potency and in vivo efficacy, together with the excellent selectivity and pharmacokinetic profiles, granted avanafil FDA approval for the treatment of male ED [232]. 

#### 6.1.3. Pyrido-Pyrimidines

Sakamoto et al. used the same general structure (**6**) reported in [231,232] and performed a cyclization between the substituents at positions 4 and 5 of the pyrimidine ring, thus developing a new series of 8-(3-chloro-4-methoxybenzyl)-8*H*-pyrido[2,3-d]pyrimidin-7-one derivatives having the (*S*)-2-hydroxymethyl-pyrrolidin-1-yl group at R^2^ similar to avanafil [233]. The potency, selectivity and in vivo efficacy of the synthesized compounds were evaluated using the same methods described in [231,232]. The standout compound of this series was compound (**10**), showing the highest PDE5 inhibitory potency (IC_50_ = 0.86 nM), the highest selectivity (selectivity factor of 2300 over PDE6) and the highest in vivo efficacy (EC_30_ = 0.85 nM) [233].

#### 6.1.4. Pyrazolopyrimidinones

The pyrazolopyrimidinone scaffold is considered a privileged scaffold when it comes to designing potent PDE5-Is, as sildenafil possesses the same scaffold. However, it retains the main disadvantage of sildenafil, which is cross-reactivity with PDE6.

Sawant et al. focused on modifying the methyl piperazine part of sildenafil, replacing it with various open-chain substitutions at the N-terminal of the sulfonamide. Substituents with an aliphatic side chain having a terminal hydroxy group or a terminal morpholine group were better than other adopted substituents. Compound **11** was the most potent PDE5 inhibitor of the series; it was twice as active as sildenafil with an IC_50_ of 1.5 nM [234]. Upon evaluating compound **11** against PDEs 1–11, it showed a similar selectivity profile to sildenafil and, most importantly, a poor selectivity over PDE6 [234]. Compound **11** exhibited 1.5 times better in vivo efficacy than sildenafil in a conscious rabbit model, however, with a poor pharmacokinetic profile (rapid metabolism by mice liver microsomes and six times higher efflux ratio in a Caco2 permeability model) [234].

In a later study, Sawant and Co. replaced the methyl piperazine moiety of sildenafil with various substituted piperidine and piperazine moieties. A piperidone moiety was optimum for PDE5 inhibitory activity, resulting in compound **12** with an IC_50_ of 0.8 nM (7 times more active than sildenafil) and a much-improved selectivity than both sildenafil and cpd. **11**, with a selectivity factor of 20 for PDE5 over PDE6 [235].


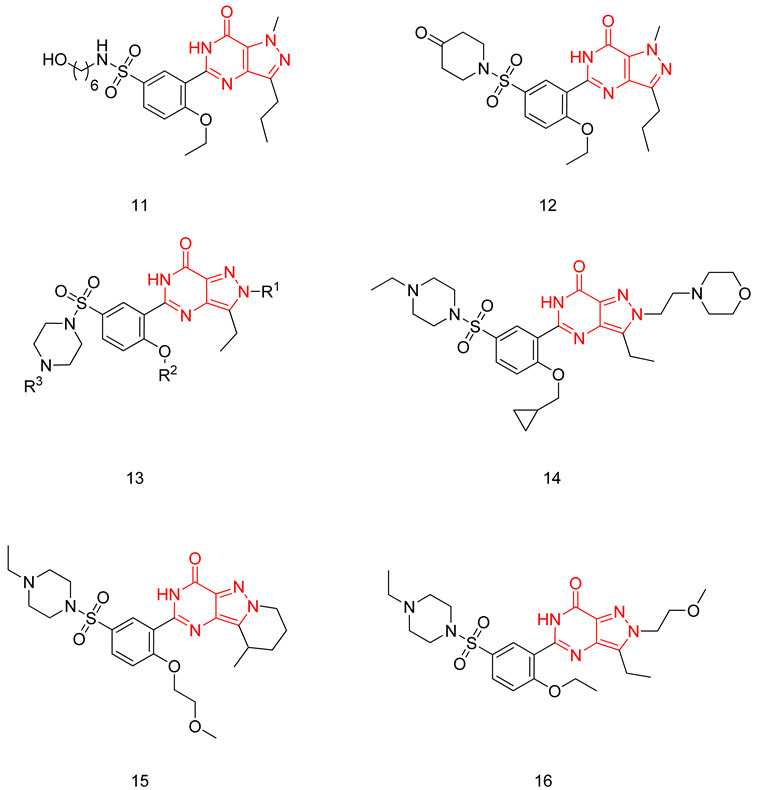


Compound **12** has revealed similar efficacy to sildenafil in maintaining penile erection in the rabbit model, with improved efflux ratio, as well as similar metabolic stability against mice liver microsomes to sildenafil [235].

Rawson et al. made a more extensive exploration of the pyrazolopyrimidines, introducing structural modifications at different positions of the scaffold, summarized in general structure **13**. The potency of the synthesized derivatives was evaluated using a PDE5 enzyme derived from the human corpus carvernosum, while selectivity was evaluated through testing against PDE6 derived from a dog’s retina [236].

At R^3^_,_ an ethyl group was optimum for PDE5 inhibitory activity. Different substituents with variations in size and chain length were all well tolerated at R^1^. However, the best substituents were either a methoxyethyl or a morpholinoethyl group. Several substituents were also well tolerated at R^2^, but using a cyclopropylmethyl moiety at R^2^ produced the most potent PDE5 inhibitor of the series (**14**) with an IC_50_ of 0.26 nM. Compound **14** was the most potent but not the most selective compound of the series. Cyclizing the N2 alkyl and the C3 to form a 3rd ring fused to the pyrazolopyrimidinone scaffold was the key structure modification to boost the selectivity for PDE5 over PDE6. Compound **15** held the highest selectivity factor over PDE6 (490) among all the synthesized analogs, with a PDE5 inhibitory activity of 1.96 nM [236]. However, it showed a relatively low oral bioavailability (18%) upon testing its pharmacokinetic properties in dogs. Compound **16** (PDE5 IC_50_ = 1.23 nM) showed the best pharmacokinetic profile among the other tested PDE5-Is with an oral bioavailability of 61%, 55% and 34% in rats, dogs and humans, respectively [236].

#### 6.1.5. Tetrahydro-β-Carbolines (THβCs)

THβCs have been extensively explored as a prominent scaffold for PDE5-Is, inspired and guided by the discovery of the FDA-approved tadalafil [237]. Abadi and co. reported the synthesis of tadalafil analogs with a tetrahydro-β-carboline-imidazolidinedione and tetrahydro-β-carboline-piperazinedione scaffolds, having different substituents at the nitrogen of the terminal ring, as well as different pedant aryl moieties at C5/C6 of the scaffold. The synthesized derivatives were tested against recombinant human PDE5 enzyme [238,239].

With a 5-bromo-2-thienyl substituent at C5/C6, the hydantoin scaffold was superior to the piperazinedione; the N-ethyl substitution was the best among all tried N-alkyl moieties, with *S* configuration at C5 essential for PDE5 inhibitory activity. The most potent compound of this series (**17**) was 53 times less potent than tadalafil (IC_50_ = 160 nM) [238], with higher selectivity than tadalafil for PDE5 over PDE11) selectivity factor of 49 vs. 13 for tadalafil) [238].

In a later study, Zheng et al. explored the use of different substituted thienyl and furyl moieties other than the 5-bromo-2-thienyl group at C5 of the THβC imidazolidinedione scaffold while keeping the other structural features of compound **17** (an *S*-configuration at C5 and an ethyl substituent at the terminal nitrogen), the 5-ethyl-2-furyl group in compound **18** showed the highest activity with equal potency to tadalafil (IC_50_ of 2.92 nM). Compound **18** was more selective than both compound **17** and tadalafil for PDE5 over PDE6 and PDE11 with selectivity factors of 43 and ˃342, respectively. As for the other PDEs, **18** showed no inhibition towards PDE1-3, PDE4 and PDE7-10 at screening doses of 20 μM, 10 μM and 3 μM, respectively [240].

In the same study, Zheng et al. employed various substituted thienyl and furyl moieties at the C6 of the THβC-piperazinedione scaffold while keeping an *S*-configuration at C6, and a methyl substituent at the terminal nitrogen of the piperazinedione ring, the 5-ethyl-thienyl group granted the most PDE5 inhibitor of the series (**19**) with an IC_50_ of 3.87 nM (equipotent to tadalafil). **19** was more selective than tadalafil, **17** and **18** for PDE5 over PDE6 with a selectivity factor of ˃258, but less selective than **18** for PDE5 over PDE11 with a selectivity factor of 70, which is still higher than both tadalafil and **17**. Similar to **18**, **19** showed no inhibition towards PDE1-3, PDE4 and PDE7–10 at screening doses up to 20 μM [240].

The in vitro vasorelaxant activities of **18** and **19** were evaluated in ^a^ rat 3rd order mesenteric arteries pre-contracted by 20 µM norepinephrine; both compounds showed a stronger vasodilatory effect than tadalafil (EC_50_ = 78 nM) with EC_50_ values of 30 and 63 nM, respectively [240].

SAR of the THβC derivatives was altered when a 4-chloro or a 4-bromo substituent was employed at C5/C6 of the scaffold; the piperazinedione scaffold was found to be superior to the hydantoin scaffold, the ethyl and butyl groups were the best among the other tried N-alkyl moieties, and an *R* configuration at C6 was essential for PDE5 inhibitory activity [241,242].

Abadi and co. explored the effect of adding a terminal amino or a hydroxyl group to the N-ethyl and N-butyl moieties of the 4-chloro and 4-bromo THβC analogs, but the attempted structural modification led to a huge reduction in PDE5 inhibitory activity. The most potent compound of the series (**20**) (IC_50_ = 100 nM) was 11 times less potent than its N-n-butyl congener (IC_50_ = 9 nM) [241] and 33 times less potent than tadalafil [239].


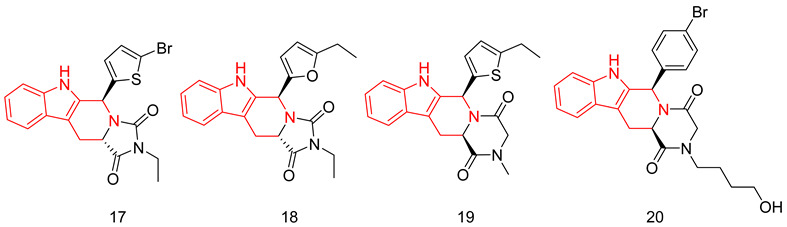


#### 6.1.6. Quinazolines

The quinazoline scaffold has been used by several researchers to obtain PDE5-Is, such as the 4-substituted variants of Watanabe [243] and the 2 & 4-substituted variants of Lee et al. [244] Gleeson and co. reported the synthesis of *N*^2^ and *N*^4^-diaminoquinazolines as PDE5-Is. At the *N*^2^ amino group, several substituted phenyls were employed, mainly a sulfonamide or an N-methylpiperazine-1-sulfonamide at either the *meta* or the *para* positions. On the *N*^4^ amino group, the substituents were either a benzyl, a substituted piperidyl or an alkyl group [245,246].

The best compound of the series(**21**) showed moderate PDE5 inhibitory potency with an IC_50_ of 72 nM (36 times less active than sildenafil) [246]. However, it showed good selectivity over PDE1, with a selectivity factor of 164 [245], as well as good efficacy when tested in an ex vivo vasodilatation model with an EC_50_ of 1.63 µM [246]. In addition to its moderate PDE5 potency, major drawbacks could be highlighted for **21**; its selectivity for PDE5 over PDE6 was less than that of sildenafil with a selectivity factor of only 4.61, besides showing high cytotoxicity in human alveolar basal epithelial cell line (ATCC CCL-185) with an IC_50_ of 11.1 µM [246].

Later, Chatturong et al. evaluated the PDE5 inhibitory potency of derivatives possessing the same scaffold against HEK293-extracted PDE5. Compounds **22** and **23** were the two most potent PDE5-Is with an IC_50_ value of 5 nM (2.5 times less potent than sildenafil) [247]. Both compounds showed a good vasorelaxant effect against isolated intrapulmonary arteries with EC_50_ values of 0.94 and 1.03 µM, respectively. Despite showing a less potent vasorelaxant effect than sildenafil (EC_50_ = 0.05 µM), their vasorelaxant effect was more selective for pulmonary arteries over the thoracic aorta. Both compounds potentiated the vasorelaxant effect of sodium nitroprusside in endothelium-denuded pulmonary arteries, and the vasorelaxant effect of both compounds was reduced upon treatment with a guanylyl cyclase inhibitor (ODQ); both results confirm that the vasorelaxant effect of both compounds is related to their PDE5 inhibitory activity. The hepatotoxicity of both compounds was evaluated in rat hepatocytes where more than 80% of the cells were viable at a test concentration of 10 µM for both compounds [247].


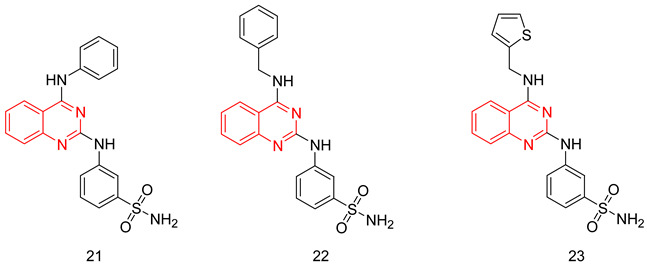


#### 6.1.7. Quinazolinedihydro-β-Carbolines

Rutaecarpine (**24**) is a quanzolinocarboline alkaloid reported to have vasodilation, anti-inflammation, and neuroprotective effects. Huang et al. introduced rutaecarpine (PDE5 IC_50_ = 1.23 µM) [248] as a lead for the development of PDE5-Is for the treatment of AD. Structural modifications were aimed at the indole part of the scaffold, with compound **25** showing the highest PDE5 inhibitory activity (IC_50_ = 86 nM). **Twenty-five** showed a better selectivity profile than sildenafil, as it showed a selectivity factor of 500 folds for PDE5 over PDE6, as well as showing no inhibition against PDE2, 4 and 9 at 500 µM. The in vivo efficacy of **25** was tested in scopolamine-induced cognitive deficit mice, where it showed relief in the learning and memory defects at a dose of 5 mg/kg [248].


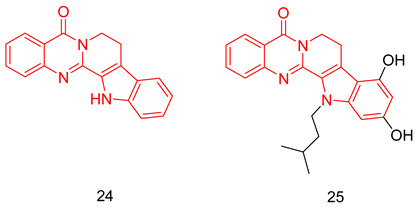


#### 6.1.8. Pyrroloquinolones

Zheng et al. succeeded in modulating the THβC scaffold into novel pyrroloquinolones with different substituted furyl and thienyl moieties at C3. The two most potent inhibitors were compounds (*S*)-**26** and (*S*)-**27** with IC_50_ values of 0.52 and 0.39 nM, respectively [249].

Both compounds showed acceptable to good oral bioavailability values (F = 24% and 66%, respectively). Moreover, both compounds showed superior in vitro vasorelaxant effects at a dose of 1 µM, as both induced almost complete relaxation in an isolated rabbit thoracic aorta contracted by norepinephrine [249]. In vivo studies in an anesthetized male New Zealand rabbits’ model showed the ability of both compounds to increase the intracavernosal pressure of electrically stimulated rabbits with ED_50_ values of 21.68 and 24.21 µg/kg, respectively, which are comparable to that of sildenafil (14.25 µg/kg) [249].


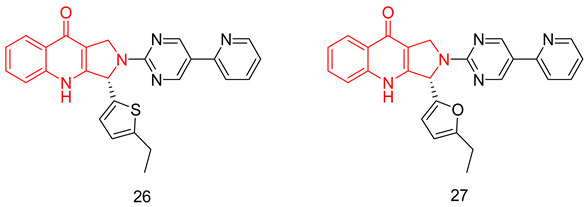


#### 6.1.9. Quinolines

Fiorito et al. reported the synthesis of 4-(3-chloro-4-methoxybenzylamino) quinoline derivatives as potent and selective PDE5-Is for the treatment of AD. All the synthesized analogs had a hydroxymethyl at C3, a benzylamino at C4 and a cyano group at C7 of the quinoline scaffold, all essential for PDE5 inhibitory activity. The PDE5 potency was evaluated using PBS PDE assay kits. The two most potent derivatives were compounds **28** and **29** with IC_50_ values of 0.27 and 0.4 nM, respectively [250]. Both compounds showed excellent selectivity profiles, showing a selectivity factor of 1256 and 12,750 over PDE6, respectively, and showing no inhibition against the other PDEs (PDE1-PDE11) at a screening dose of 10 μM [250].

Compound **28** was then chosen to be further evaluated in vivo. Upon testing compound **28** in a male BALB/c model, it exhibited a good pharmacokinetic profile, reaching the maximum plasma concentration in 30 min, besides showing a fast distribution to the brain as the *T_max_* values in the brain and plasma were similar. Compound **28** was able to elevate the cGMP levels in the hippocampus of adult mice after administration of a 3 mg/kg dose followed by a foot shock after 30 min. Moreover, **28** was tested in mice treated with oligomers of Aβ_42_ that are known to induce loss in memory and hippocampal long-term potentiation and were found to restore the long-term potentiation effect, as well as treating the behavioral defects in mice caused by the loss of memory [250].


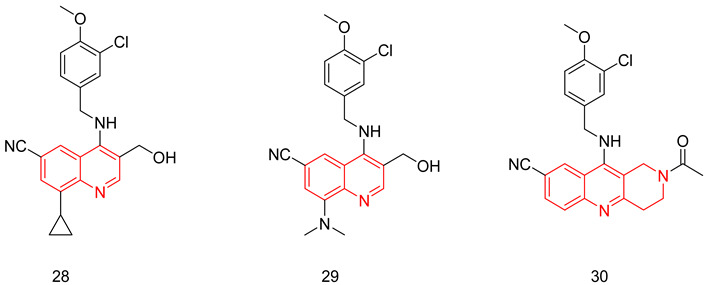


#### 6.1.10. Tetrahydrobenzo[b][1,6]Naphthyridine

In a later study, Fiorito et al. further optimized their quinoline derivatives to improve their water solubility. The next scaffold was obtained by locking the rotatable bonds of the hydroxymethyl group of compounds **28** and **29** into a ring to give tetrahydrobenzo[b][1,6]naphthyridine scaffold [251]. This rigidification strategy led to the discovery of compound **30,** which elicited higher potency against PDE5 (IC_50_ = 0.056 nM) and improved aqueous solubility. Additionally, compound **30** exhibited more than 500-fold selectivity for PDE5 vs. PDE6. However, the selectivity profile vs. other PDE isoforms, especially PDE11, was not presented. In a mouse model of AD, **30** improved learning and memory impairments by raising cGMP levels in the hippocampus. The very low microsomal metabolic stability was one of the major drawbacks of this very potent class of compounds that needs further optimization [251].

#### 6.1.11. Chromenopyrrolones

Luo group reported the synthesis of chromeno [2,3-c]pyrrol-9(2*H*)-ones as PDE5-Is with a general structure (**31**) [252]. From the inhibitory activity data of the compounds, the following SAR could be concluded: PDE5 inhibition is hugely affected by the substitution at R^3^ and R^4^; at R^3,^ using 5-membered heterocycles was preferred to substituted phenyl moieties, biphenyl, and naphthalene rings while the best substitution at R^4^ was a *p*-hydroxy benzyl moiety. The two most potent inhibitors were compounds **32** and **33**, with IC_50_s of 17 and 18 nM, respectively, against PDE5 [252].

Compound **32** was further tested against PDE1, 4, 7, 8, 9 and 10. Despite showing good selectivity for PDE5 over the tested kinases (IC_50_s ˃ 750 nM), its overall selectivity couldn’t be judged as its activity against the two most common off-targets, PDE6 and PDE11 was not evaluated. One drawback of compound **32** is the relatively weak pharmacokinetic properties with an oral bioavailability of 4.9% [252].

In a later study, **32** was further optimized using the structure-based approach, where the thiophane ring at C1 was converted to thiazole, aiming at establishing a key bidentate H-bond interaction with Gln817 involving the thiazole nitrogen and the NH of pyrrole (colored in blue). This successfully led to compound **34** with an IC_50_ of 5.4 nM. It is worth mentioning that the other six and five-membered heterocycles at C1 did not lead to the same improvement in potency. In order to improve the metabolic stability and PK properties, the 4-hydroxybenzyl group in **34** was replaced with benzodioxole moiety to give compound **35** with a highly improved PK profile (F = 63.4%). In addition, **35** exhibited good drug-like properties, such as human liver microsomal stability, low cytochrome inhibition, low hERG inhibition, and pharmacological safety. When tested in the PAH in vivo model, **35** exhibited higher efficacy than sildenafil. Generally, **35** exhibited good isoform selectivity for PDE5. However, it had only selectivity indices of 10 and 27 for PDE6 and PDE10A, respectively, while selectivity against PDE11 was not presented by the authors [253]. Finally, the Luo group crowned their efforts in developing this class by further synthesis of nineteen analogs, which all showed IC_50_ values towards PDE5 of less than 10 nM [254]. In these analogs, compound **35** was further optimized on two stages, (i) through adding mono/di substituents at positions 5, 6, 7 and 8, yielding several more potent compounds like compounds **36** and **37** (IC_50_ = 1.41 and 1.05 nM, receptively). (ii) cocrystal of **34** with PDE5 catalytic domain (PDB code 5ZZ2, see binding modes section)) has guided the synthesis of compound **38** with a sub-nanomolar IC_50_ against PDE5 (0.32 nM). **Thirty-eight** exhibited a high selectivity index (SI) vs. many other PDE isoforms; however, compared to compound **35**, the SI for PDE5 vs. PDE6 was compromised from 10 to 4 (comparable to sildenafil), while SI vs. PDE11 was disclosed to be 122 (SI of tadalafil = 20). Similar to compound **35**, **37** exhibited good drug-like properties; the higher in vitro potency was correlated to a higher pharmacodynamic effect in vivo than **33** and sildenafil in the PAH model [254].


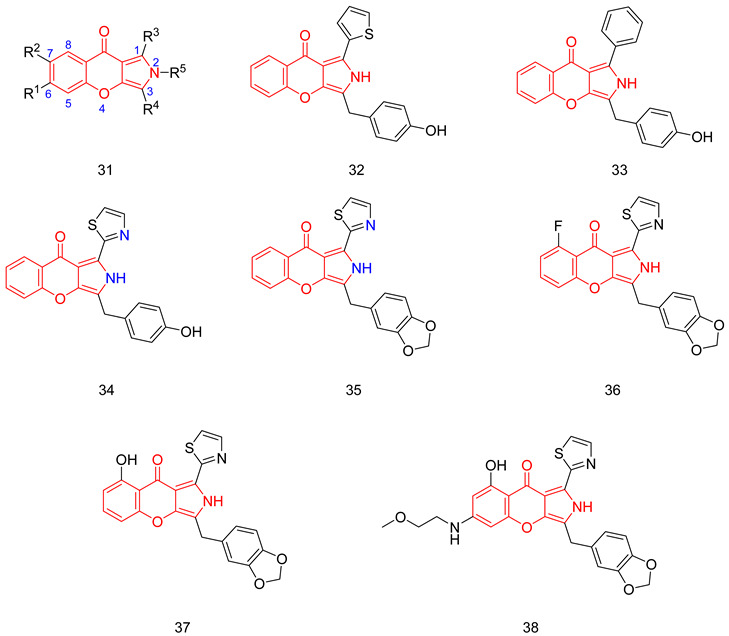


#### 6.1.12. Azepinoindolones

Based on the structures of tadalafil and the chromenopyrrolone derivative **35**, Luo and co-workers used a free energy perturbation-guided scaffold hopping strategy to identify **39**; a 2,3,4,6-tetrahydro-1*H*-azepino[5,4,3-cd]indol-1-one derivative with a PDE5 inhibitory activity of 55 nM. Structural modifications on **39** led to the discovery of **40,** a potent PDE5 inhibitor with an IC_50_ of 8.3 nM. **40** showed a 20 folds better selectivity for PDE5 over PDE11 than tadalafil, but almost the same selectivity for PDE5 over PDE6 as sildenafil. No significant inhibition was observed against other PDEs [255].

The in vivo efficacy of **40** was evaluated using a monocrotaline-induced pulmonary arterial hypertension rat model. Treatment of rats with a 2.5 mg/kg intraperitoneal dose of **40** for three weeks gave an effect comparable to that produced upon oral administration of a 10 mg/kg dose of sildenafil citrate for the same period. Furthermore, no acute toxicity was observed on the first day upon oral administration of a 1.5 mg/kg dose of **40** in male rats [255].


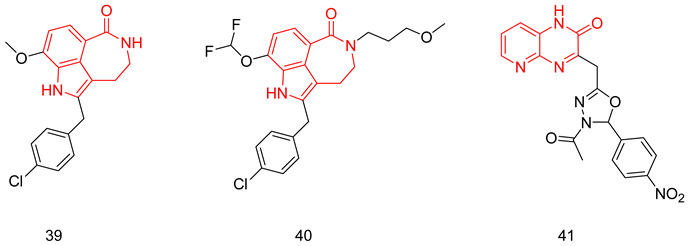


#### 6.1.13. Pyridopyrazinones

Pyridopyrazinone-based derivatives have a wide spectrum of biological activities, such as CRF-R1 (corticotropin-releasing factor receptor 1) antagonists, PI3K inhibitors and antiproliferative agents [256]. In 2009, Pfizer Global R&D reported a new series of tri-substituted pyridopyrazinone derivatives as PDE5-Is [257,258]. This encouraged Amin et al. to evaluate their previously reported anti-cancer agents bearing a mono-substituted pyrido[2,3-b]pyrazinone scaffold against PDE5. In silico studies and in vitro biological testing disclosed compound **41** as the most potent PDE5 inhibitor, with an IC_50_ of 18.13 nM (9 times less potent than sildenafil). Selectivity testing against other members of the PDE family and in vivo studies were not reported for **41** [256].

#### 6.1.14. Thienopyrimidines

Abadi and coworkers reported a series of 4-substituted thienopyrimidines fused to cyclopentene or cycloheptene. Several amnio substituents were tried at position 4, including aryl/acetyl/methyl piperazino groups, cyclohexlmethyl amino and arylhydrazones. Among more than 50 presented analogs, compounds **42** and **43** showed submicromolar potency against PDE5 with IC_50_ values of 0.42 and 0.19 µM, respectively. Selectivity was only presented vs. PDE7 and PDE9, where the two compounds weakly inhibited both enzymes at 25 µM [259].


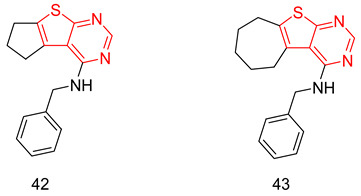


#### 6.1.15. PDE5 Allosteric Inhibitors

Targeting non-active site regions, which are less conserved, may offer a better chance to obtain selective PDE5-Is. This will be discussed in the next section.

##### Evodiamine Derivatives


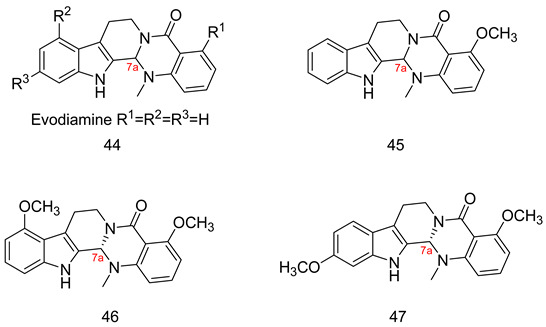


Evodiamine (**44**) is a natural product that was shown to inhibit PDE5 with an IC_50_ of 2.1 µM. In their efforts to discover PDE5 allosteric inhibitors with improved selectivity profiles, the Luo group characterized allosteric pockets 468 Å^3^ on the PDE5 catalytic domain using Allofinder [260]. After virtual screening of their natural product library followed by in vitro enzyme assay, compound **45** and its *S* enantiomer were identified as PDE5-Is with IC_50_ values of 340 and 110 nM, respectively, with 5–10 fold better selectivity profile vs. PDE6 than sildenafil. A small library of compounds was synthesized guided by molecular docking to reduce synthetic efforts. Regarding the chiral center at position 7a, the activity of the *S* isomer was higher than the racemate, while the *R* enantiomer was much less active. At R^1^, the methoxy > hydroxy > hydrogen regarding the potency. Having a 2nd methoxy group at R^2^ or R^3^ further increased the potency to yield compounds **46** and **47** with IC_50_ values of 35 and 42 nM, respectively. However, **47** exhibited better oral bioavailability (14%) compared to **46** (1%).

Enzyme kinetics revealed non-competitive behavior for **46**; this is unlike all the previously reported PDE5-Is, which were shown to be substrate-competitive. Additionally, a cocrystal of **46** with PDE5 catalytic domain (PDB 6VBI) showed that the compound binds to a novel allosteric pocket on the catalytic domain, designated as EVO pocket. (See the crystal structures/docking section). Due to their allosteric nature, **46** and **47** exhibited excellent selectivity profiles with more than 570-fold selectivity over other PDEs except for PDE10 (20–30 folds). Compound **47** exhibited comparable in vivo efficacy to sildenafil in the pulmonary hypertension mouse model.

##### Trisubstituted Pyrazolines

Celecoxib (**48**), the selective COX-2 inhibitor, was shown to have an off-target activity towards PDE5 with an IC_50_ of 37 µM. Unlike the approved PDE5-Is, its PDE5 inhibitory activity was found to be against the full-length enzyme but not the PDE5 catalytic domain, indicating that its activity is strictly dependent on the presence of PDE5 regulatory domain, giving promise to develop a class with higher selectivity than the conventional PDE5-Is [261]. Abdel-Halim et al. reported a selective optimization of side activities (SOSA) approach to enhance the PDE5 inhibitory activity of celecoxib and abolish the COX-2 inhibition. The major structural variations introduced to celecoxib were (i) the replacement of the sulfonamide group with a carboxylic acid group, (ii) using the non-planar pyrazoline core instead of the pyrazole, (iii) using the *t*-Bu instead of the trifluoromethyl group and (iv) trying different substituents at the 5-phenyl. This resulted in compound **49** with an IC_50_ of 2 µM against PDE5 and diminished activity against COX-2 [261]. Further structure-based optimization via systematic modifications for **49** led to compounds **50** and **51** with an IC_50_ of 4 and 1 nM, respectively. Incorporation of the carboxylic acid functional group in an amide bond with methyl piperazine and difluorination at *meta* or *ortho* and *meta* positions of the 5-phenyl were key changes that achieved this huge boost in PDE5 inhibition. **Fifty-one** exhibited an unprecedented selectivity profile, almost approaching 15,000 fold toward all PDE isoforms, including PDE6 and PDE11 [261]. Similar to celecoxib, **51** was only active against the PDE5 full-length enzyme and not the PDE5 catalytic domain, justifying the unprecedented selectivity profile. Until now, only the PDE5 catalytic domain could be crystalized with several inhibitors. Thus, a cocrystal of **51** with a PDE5 full-length enzyme was not yet feasible. As expected, the study of PDE5 enzyme kinetics with **51** ruled out the competitive mode of inhibition, with no clear tendency towards a non- or uncompetitive mode of inhibition. The authors suggested that, presumably, **51** has a mixed mode of inhibition, with a preferred binding to the apoenzyme or to the substrate-bound form. As for the binding site, it might be located in the interface between the active site of PDE5 and the GAF-B domain, or it might be an allosteric binding to the regulatory domain with allosteric modulation of the active site. Keeping in mind that till now, the full-length PDE5 enzyme could not be crystallized, it remains an important question to be answered: Where do these intriguing pyrazolines exactly bind?


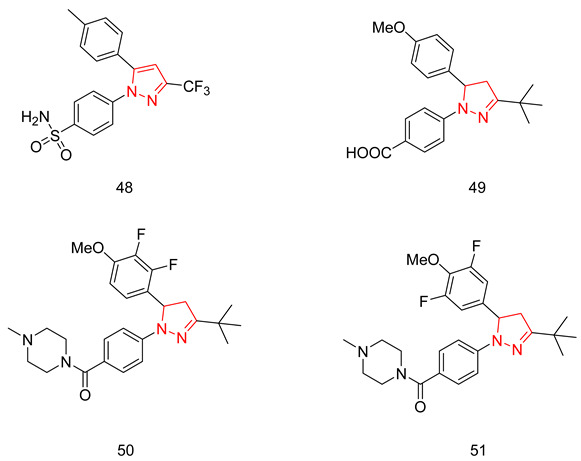


### 6.2. PDE5 in the Context of Dual Inhibitors

#### 6.2.1. Compounds with Dual PDE5 and HDAC Inhibitory Activities

PDE5 and HDAC (histone deacetylase) were reported as therapeutic targets in AD. Oyarzabal and Co. aimed at designing dual PDE5 and HDAC inhibitors as multitarget directed ligands for the treatment of AD, where the complex etiology of the disease suggests higher efficacy than the classical ‘one molecule-one target approach’. Despite accumulated evidence that targeting HDAC6 specifically is beneficial for treating AD, Oyarzabal and co. designed three classes of inhibitors with different HDAC inhibitory profiles: class A, having a pan-HDAC inhibitory activity; class B, having selectivity for HDAC6 over class I HDACs; and finally, class C, having selectivity for class I HDACs over HDAC6. For these inhibitors to be successful, they were planned to have moderate inhibition for HDAC class I (HDAC1, 2, 3 and 8) to avoid the possible toxicity but potent PDE5 inhibition to provide the synergistic effect needed for a cellular and an in vivo functional response, as well as CNS-penetration. In their studies, structural modifications were made to replace the piperazine sulfonamide part of sildenafil and vardenafil with groups that have a terminal zinc binding group ZBG (a hydroxamic acid or an ortho-aminoanilide moiety), which is essential for HDAC inhibition. They also employed similar modifications at the piperidinedione nitrogen of tadalafil [262,263,264,265].

In their first report, Oyarzabal and Co. focused on developing class A inhibitors using the sildenafil core, together with a wide range of substituents having a terminal hydroxamic acid group (a ZBG essential for HDAC inhibitory activity) and linked to the 5′position of the phenyl ring of sildenafil via a carbon, nitrogen, or an oxygen atom [262].

Despite being a 7-fold less potent PDE5 inhibitor than sildenafil, compound **52** (CM-414) was the best pan-HDAC inhibitor. The cytotoxicity of **52** was evaluated in THLE-2 cells and primary neuronal cultures of glia cells and showed moderate cytotoxicity in THLE-2 cells and low cytotoxicity in glia cells, with an LC_50_ of 7.2 µM and 17.7 µM, respectively [262].

**52** was further studied in vivo, where it increased the acetylation of histone by 98% and increased the phosphorylation of CREB by 148% in the hippocampus of mice 30 min post a 40 mg/kg intraperitoneal injection [262]. It was reported in another related study that chronic treatment of Tg2576 mice with **52** resulted in a huge decrease in the brain Aβ and pTau levels through favoring the inactive form of GSK3β, reverted the decrease in dendritic spine density on hippocampal neurons, and reversed the memory impairment in mice through inducing the expression of genes related to synaptic transmission [266]. Furthermore, **52** succeeded in decreasing the expression of fibrogenic markers and collagen deposition in Mdr2-KO mice (a clinically relevant model of liver inflammation and fibrosis), impeding the progression of chronic liver disease in this type of mice [267].

Despite showing a good functional response both in vitro and in vivo, some drawbacks of **52** could be highlighted: its moderate PDE5 potency in comparison to other synthesized derivatives; it was highly potent against PDE6 (IC_50_ = 2.6 nM), and finally, its poor in vitro pharmacokinetics, attributed to its low permeation (P_e_ value = 15.7 nm/s in a PAMPA assay), as well as its high efflux ratio (41.3 in a Caco-2 permeability assay) [262].

The vardenafil-matched pair of **52** was reported in another study carried out by Oyarzabal and co [263]. Despite being a more potent PDE5 inhibitor than **52**, compound **53** showed moderate cytotoxicity upon its evaluation in THLE-2 cells, primary neuronal cultures of glia cells and peripheral blood mononuclear cells (PBMCs) with LC_50_ values of 9.37 µM, 6.1 µM and 6.2 µM, respectively. It can be thus concluded that **53** has a narrow therapeutic window, given the fact that 400 nM was needed to elicit a significant in vitro cellular response. Moreover, **53** showed poor in vivo efficacy, producing only a 25% increase in the levels of phosphorylated CREB in the hippocampus of mice after 1 h of a 40 mg/kg intraperitoneal injection [263].

Following this study, Oyarzabal and co. focused on developing inhibitors of class B, using both the sildenafil and vardenafil cores. Different phenyl, substituted phenyl, thienyl and furyl moieties linked to a terminal hydroxamic acid group were employed at the 5′position of the phenyl ring of the core. Compound **54** was the most potent HDAC6 inhibitor, with excellent selectivity over class I HDACs, in addition to having potent PDE5 inhibition. **Fifty-four** showed moderate cytotoxicity in THLE-2 cells and low cytotoxicity in primary neuronal cultures of glial cells, with an LC_50_ of 6.81 µM and 46.3 µM, respectively [265].


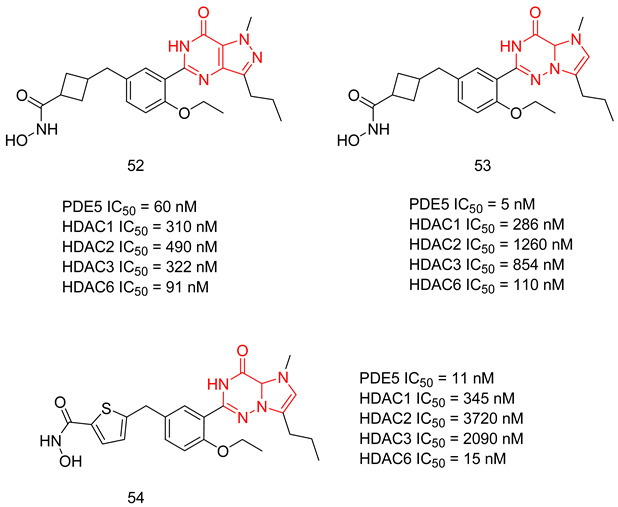


Despite showing a good functional response in vitro, **54** was able to increase the level of phosphorylated CREB by only 48% in the hippocampus of mice after 30 min of a 40 mg/kg intraperitoneal injection. In addition, no significant improvement in the memory of Tg2576 mice was observed following a two-week treatment with **54** [265].

Finally, Oyarzabal and co. aimed at designing inhibitors of class C, using both the sildenafil and vardenafil cores. A wide range of phenyl and cycloalkyl substituents were employed with an *ortho*-aminoanilide moiety as the ZBG instead of the hydroxamic acid group, a structural modification that led to the masking of the HDAC6 inhibitory activity. Compound **55,** having a vardenafil core, was the most potent dual PDE5/class I HDAC inhibitor [263]. The second-best class I HDAC inhibitor was compound **56**, which, despite having a moderate PDE5 inhibitory activity, was the compound of choice for further in vivo testing [264]. **Fifty-six** showed a low cytotoxicity profile, with an LC_50_ of 11,700 nM in THLE-2 cells, while no effect was observed in PBMCs at a screening dose of 100 µM. Despite showing an excellent pharmacokinetic profile, with a P_e_ value of 82.9 nm/s in the PAMPA assay and a low efflux ratio (0.86), **56** failed to produce a significant reversing effect for the memory impairment of Tg2576 mice after administration of a 20 mg/kg dose for two weeks [264].


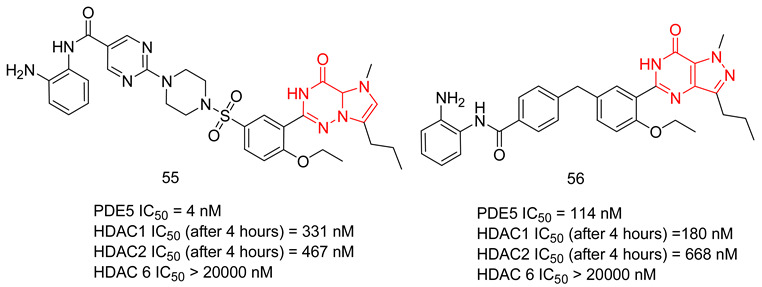


Upon comparing the three classes of inhibitors reported by Oyarzabal and co., inhibitors of class A employed on a sildenafil core provided the best pharmacological profile needed for AD treatment, as it was the only inhibitors class whose stand-out compound, despite having poor pharmacokinetics, showed memory restoration in mice AD model.

A few substituents were employed by Oyarzabal and Co. on the tadalafil core, specifically on the terminal nitrogen of the piperazinedione ring of tadalafil. The reported compounds were either inactive or much less active than their sildenafil and vardenafil matched pairs [263,264].

As an anticancer agent, tadalafil is usually not used solely but rather in combination with other chemotherapeutic agents to enhance its cytotoxicity against numerous types of cancer cells [76]. HDAC inhibitors could be regarded as one of the most important chemotherapeutics that could be used in combination with tadalafil for cancer treatment. Noteworthy, pan-HDAC inhibitors like vorinostat (SAHA), belinostat and panobinostat were approved by the FDA for the treatment of some types of hematological cancers [268].

As mentioned previously, the incorporation of a terminal amino or a hydroxyl group to the N-ethyl and N-butyl moieties of the 4-chloro and 4-bromo THβC analogs by Abadi and co. led to a sharp decrease in the PDE5 inhibitory activity [239]. Therefore, in a more recent study, Abdel-Halim and co. incorporated a terminal ZBG (carboxylic acid or a hydroxamic acid group) instead and manipulated the spacer length between the nitrogen of the piperazinedione and the terminal ZBG. Inhibitors with a terminal carboxylic acid group were only active against PDE5, while those with a stereochemistry of 6*S* and 12a*S* were only active against HDAC. The most potent dual inhibitor was compound **57,** with an IC_50_ of 46.3 against PDE5 and a pan-HDAC IC_50_ of 14.5 nM [269].

**Fifty-seven** had a moderate in vitro cellular potency against three types of colon cancer cell lines (HT-29, HCT-116 and SW-620), with GI_50_ values of 12.35, 7.19 and 11.79 µM, respectively. **57** was also tested against several types of cancer cell lines, showing a high cellular potency (<3 µM) against Molt 4 (acute lymphoblastic leukemia), Sup-T1 (T-cell lymphoblastic lymphoma), K562 (chronic myelogenous leukemia), as well as T47D (breast cancer cells), where it could induce apoptosis in Molt-4 cells. NCI-60 human tumor cell line screen showed a high selectivity of **57** for leukemia and solid tumor cell lines. **Fifty-seven** showed a good therapeutic window, as it did not show any significant inhibitory effect in the non-cancer cell line CCD966SK with a GI_50_ of 28.2 µM [269].


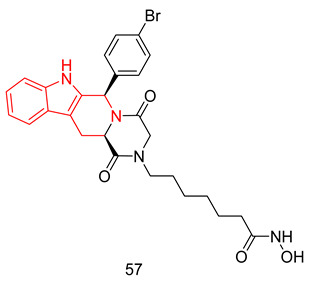


#### 6.2.2. Compounds with Dual PDE5 and AchE Inhibitory Activities

Four of the clinically approved drugs against AD are AchE inhibitors. AchE inhibition has been shown to improve memory and cognitive functions in AD patients; therefore, designing MTDLs towards AchE and another target involved in AD pathology would produce a more significant reduction in AD symptoms. Mao et al. reported the synthesis of tadalafil analogs with varying substituents at the nitrogen of the terminal piperazinedione ring as dual AchE/PDE5-Is for the treatment of AD [270].

The two most interesting compounds of the series (**58** and its diastereomer **59**) were the most potent derivatives against aChE with IC_50_s of 36 and 32 nM with PDE5 inhibitory IC_50_s of 150 and 1530 nM, respectively. Further evaluation of **58** and **59** revealed their ability to cross the BBB upon testing them in a parallel artificial membrane permeation assay. **58** and **59** could inhibit PDE5 in vivo as they significantly enhanced the phosphorylation of CREB. Finally, at a dose of 10 mg/kg, **59** was more effective than **58** at improving the cognitive functions of scopolamine-induced cognitive deficit mice [270].


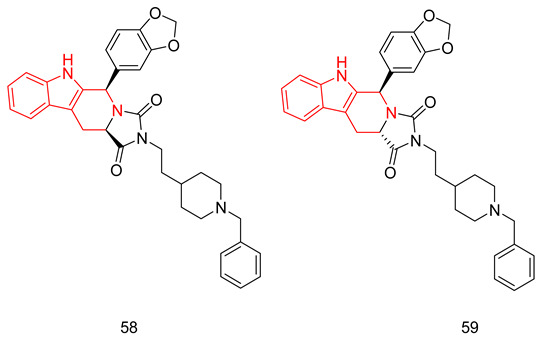


#### 6.2.3. Dual PDE5 Inhibitor and NO Donor

Topical or systemic application of sildenafil has been shown to be beneficial for wound healing through increasing cGMP levels. The significant role of NO in wound repair has been clearly observed in mice deficient for inducible or endothelial NO synthase, where they showed delayed wound closure and impaired angiogenesis. The in vitro and in vivo effects of NO donors and PDE5-Is on the different cell types involved in wound repair, as well as the potential additive effect of NO supplementation and PDE5 inhibition, were not tested until Greenwald et al. reported the synthesis of TOP-N53 (**60**), a dual-acting NO donor and PDE5 inhibitor, having wound healing effects in both normal mice and mice with diabetes mellitus [271].

**Sixty** showed a PDE5 inhibitory potency of 1.6 nM, and it significantly increased the levels of NO in human keratinocytes after 24 h from the treatment with a 10 µM dose of **60** and up to 72 h [271].

The wound healing effects of **60** were tested in both healthy mice and mice with diabetes mellitus. Treatment with **60** led to the closure of 26% of the wounds in normal mice on the 5th day of treatment, where it induced keratinocyte proliferation, angiogenesis, and collagen maturation in wounded skin without enhancing the normal wound inflammatory response. A similar effect was seen in mice with diabetes mellitus, where **60** promoted re-epithelization, as well as angiogenesis, without enhancing the normal inflammatory response [271].


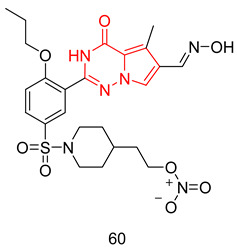


#### 6.2.4. Compounds with Dual PDE5 and Topoisomerase 2 Inhibitory Activities

Abadi and co. tried to combine the anticancer properties of PDE5 and Topoisomerase II inhibitors in a single molecule through the synthesis of several 9-benzylaminoacridine derivatives. They presented dual PDE5-Topoisomerase 2 inhibition as a potential strategy against colorectal cancer. The most potent PDE5 inhibitor of the series **61** (IC_50_ = 0.83 µM) exhibited lower growth inhibitory activity against colorectal cells (HCT-116). On the other hand, the three most potent topoisomerase two inhibitors, **62, 63** and **64,** showed low micromolar PDE5 inhibitory activity and significant growth inhibitory effects against HCT-116 cells. However, **64** was shown to have less selective anticancer activity, where it showed a relatively high growth inhibition against the non-malignant dermal fibroblast CCD-966SK cells (IC_50_ = 4.67 µM) [272].


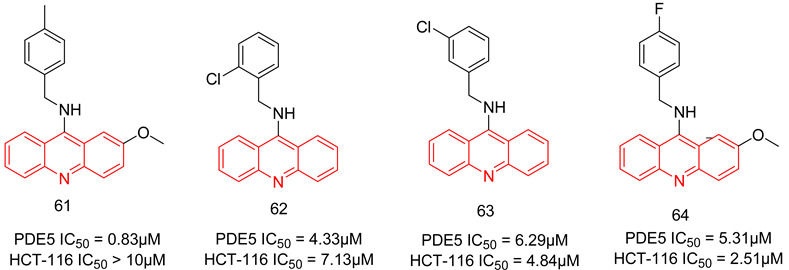


### 6.3. PDE5 Inhibitors for Radiodiagnosis

Positron emission tomography (PET) is one of the most sensitive in vivo molecular imaging modalities that are based on utilizing radiotracers labeled with short-lived positron-emitting radionuclides [273]. Lately, there is growing interest in the design and biological evaluation of PDE5-specific PET radiotracers, which would facilitate the non-invasive evaluation of PDE5 expression levels in vivo. Using PDE5 radiotracers with optimal pharmacokinetic profiles in combination with high accuracy would likely address multiple issues facing clinical investigators, such as (i) providing valuable diagnostic information regarding the localization, extent and severity of impairments and/or deregulation of the cGMP/PDE5 pathway, thus permitting an early identification of patients that would benefit from treatment with PDE5-Is, (ii) quantification of changes in PDE5 expression during disease progression, and (iii) assessment of occupancy by PDE5-Is in target tissues enabling physicians to tailor optimal dose and dosage regimens on an individual basis [274,275,276].

Despite the availability of many specific and high-affinity PDE5-Is, only a few radiotracers have been evaluated for PET imaging of this enzyme until now. The first PDE5 radiotracers were reported by Chekol et al., who developed the carbon-11 (**65**) and fluorine-18 labeled (**66**) vardenafil-based PDE5 radioligands [274]. Both radiotracers exhibited high retention in the lungs, and their specific inhibition of PDE5 was proven via a pre-blocking study in mice by tadalafil. However, none of those ligands demonstrated significant brain uptake [274]. A later report by the same group revealed that a pyridopyrazinone based ^18^F-labeled derivative (**67**) exhibited the highest PDE5-specific retention in the lungs of wild-type mice and in the myocardium of transgenic mice with cardiomyocyte-specific PDE5 over-expression. Although (**67**) readily entered the brain, its radioactivity uptake was found not specific toward the PDE5 [275]. More recently, a 4(3*H*)-pyrimidinone compound (**2**) developed for the treatment of PAH was ^11^C-radiolabeled at the *N*-methyl of its piperazine ring. However, the authors did not perform a biodistribution study or an in vitro assessment of tracer inhibitory activity to ensure that the radiotracer’s binding ability to PDE5 was unaffected [277]. Very recently, a ^14^C-radiolabeled derivative of (**2**) was used to assess its pharmacokinetics where (**2**) exhibited rapid absorption in humans (*T_max_* = 0.67 h) and t_1/2_ of 9.9 h, besides extensive metabolism into 22 metabolites in human plasma, urine and feces [278].

Several studies by the research group of Liu, Wenzel and coworkers have focused on the development of fluorinated quinoline derivatives as brain-specific PDE5 tracers. However, all promising candidates showed high non-specific retention in the brain besides their fast metabolism in vivo, forming brain penetrable radio metabolites [273,276,279]. The challenging design of PDE5-specific radiotracers in the brain is likely attributed to the substantially low PDE5 expression in the brain with only nanomolar density. Accordingly, a radioligand with at least sub-nanomolar PDE5 potency is needed for the quantification of brain PDE5 [280]. This belief guided Dong et al. to ^11^C-radiolabel the *O*-methyl of the previously reported picomolar potent PDE5-Is (**28** and **30**) for the treatment of AD. However, neither good brain penetration of those radiolabeled tracers nor their specific PDE5 binding in vivo was validated by authors, requiring further preclinical investigations [280].

In 2022, avanafil (**9**) was successfully labeled with iodine-125 via an electrophilic substitution reaction of its methoxy-activated aromatic ring. An in vitro stability study and evaluation of the tracer’s PDE5 inhibitory activity, in addition to biodistribution and clearance studies in rat models of ED, have verified the applicability of radiolabeled avanafil as a promising tracer for ED [281].


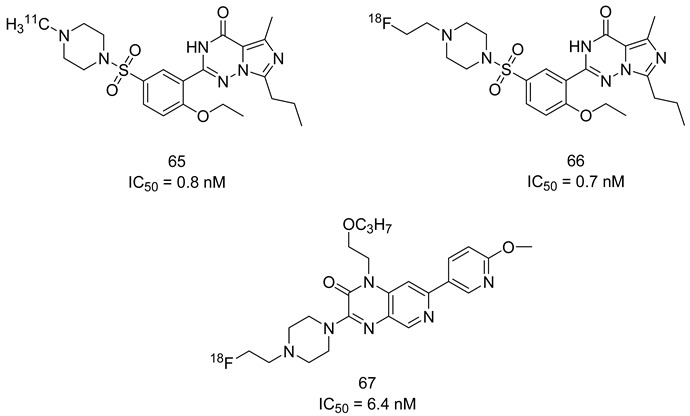


## 7. Binding Modes of PDE5 Inhibitors

### 7.1. The PDE5 Active Site

The catalytic domain of PDE5 has three helical subdomains: an N-terminal cyclin-fold region, a linker region, and a C-terminal helical bundle. The active site of PDE5 is located at the center of the C-terminal helical bundle domain, and it can be divided into five subsites: [241,282]

1. A metal-binding site (M site) that contains Zn^+2^ and Mg^+2^ ions, together with several aspartate and histidine residues.
2. A core pocket (Q pocket) lined by Gln817, Phe820, Val782, and Tyr612.
3. A hydrophobic pocket (H pocket).
4. The Q2-pocket, which is lined by Phe786, Phe787, Leu804, Ile813, Met816.
5. The Lid region consists of Tyr664, Met816, Ala823 and Gly819.

A deep insight into the X-ray cocrystal structures of PDE5 enzyme with inhibitors resolved to date provides comprehensive data about the key interactions involved in inhibitors binding and reveals that inhibitors binding to PDE5 active site mainly adopt either a sildenafil/vardenafil-like binding mode or a tadalafil-like binding mode (summarized in Table 1).

Sildenafil is stabilized in the active site of the PDE5 through (a) a bidentate hydrogen bond with the amide group of Gln817, (b) hydrophobic interactions of the pyrazolopyrimidinone core with the side chains of Val782, Leu785, Tyr612, and Phe820, including a face-to-face pi–pi interaction with the phenyl ring of Phe820. In addition, the N2 of the pyrazole forms water-mediated interactions with the Zn^+2^ in the M site and the side chain of Tyr612. Moreover, the ethoxyphenyl group of sildenafil fits into the Q2 pocket, and finally, the methylpiperazine group of sildenafil is embedded in the L-region [282]. Vardenafil shows a very similar binding mode to sildenafil where no extra interactions are observed for the ethylpiperazine of vardenafil in comparison to the methylpiperazine moiety of sildenafil [282].

The binding mode of tadalafil shows some similarities to that of sildenafil, where the beta-carboline core of tadalafil forms CH-pi interactions with Phe820 and Val782, as well as hydrophobic interactions with Val782 and Tyr612. Moreover, the benzodioxole moiety resides in the Q2 pocket, similar to the ethoxyphenyl group of sildenafil, showing interactions with the residues lining the pocket (mainly Phe786 and Leu804). However, three major differences could be highlighted between the binding modes of sildenafil and tadalafil: (a) tadalafil binds to the conserved Gln817 residue via a monodentate hydrogen bond; (b) tadalafil shows no interactions with the M site; (c) tadalafil shows no interactions with the L-region [241,282].

### 7.2. The Evo Pocket

Zhang et al. identified a novel site for the binding within the catalytic domain of PDE5 other than the competitive binding site targeted by sildenafil, vardenafil and tadalafil, designated as the EVO pocket. The EVO pocket is located at the back of the active site and is isolated from it by Tyr612, His 613, His617, and Leu781. It is composed of a hydrophobic wall (Phe564, Ile778, Leu781, and Tyr612), a polar wall (Asp563, Arg616, and Asn620), and a polar bottom (Asp764 and His617) [260].

Compound **46** reported by Zhang et al. as a potent allosteric inhibitor for PDE5 with an IC_50_ of 0.035 µM was co-crystallized with the PDE5 enzyme (PDB ID: 6VIB), and it was shown to be anchored in the EVO pocket via (i) three H-bonds with the side chains of Asp563, Asn614, and His617, (ii) a water-mediated H-bond with Ala767 and Asp764, in addition to (iii) numerous van der Waals interactions with residues Ile778, Leu781, and Phe564 (Table 1) [260].

## 8. Recent Update on Clinical Trials Involving PDE5 Inhibitors

A plethora of preclinical and clinical studies were conducted over the past 20 years to evaluate the role of PDE5-Is either as a single treatment or in combination therapy for various FDA-approved or emerging clinical conditions. These studies have been extensively discussed in several previous reviews [17,23,76,122,123,283,284,285,286,287]; thus, we herein present an update of the clinical trials status of PDE5-Is in the past five years (summarized in Table 2). Focus was mainly directed to registered trials in ClinicalTrials.gov (accessed on 1 August 2023) that are complete with published results. Moreover, a summary of clinical trials that are currently open (recruiting or soon to commence recruitment) or ongoing is presented in Table S1 (Supplementary Materials), providing a comprehensive overview of the current status of drug discovery efforts involving PDE5 as a therapeutic target. It is worth noting that current clinical trials assessing PDE5-Is in CVS diseases are mainly focused on right ventricular dysfunction, congenital heart disease, or cystic fibrosis, so further trials are yet needed to explore the efficacy of PDE5-Is on cardiac outcomes in myocardial infarction, coronary artery disease, heart failure, and ventricular arrhythmia. Similarly, the potential utility of PDE5-Is as reno-protective agents in AD or in non-ED urological diseases requires further support by carefully designed dose-dependent and time-course animal and clinical studies. On the other hand, a plausible number of ongoing trials are investigating the effects of PDE5-Is in unconventional clinical conditions, such as obesity, retinitis, scleroderma, liver fibrosis, depression, Duchenne muscular dystrophy and fetal hypoxia Table S1 (Supplementary Materials). Accordingly, revealing novel clinical applications for PDE5-Is could be anticipated in the near future.

## 9. Conclusions

The evidence presented in this review underscores the pivotal role of PDE5-Is as disease-modifying agents, not only in the treatment of erectile dysfunction and pulmonary hypertension but also in the treatment of a wide array of other diseases, ranging from cognitive impairments to immune disorders and beyond. Most of the emerging uses of PDE5-Is arise from their abilities to modulate the level of the universal cellular secondary messenger cGMP with its implication in various pathological and physiological conditions. This is expected to provide new approved and off-label uses for this class of medications.

The primary challenge in the development of PDE5-Is as potential drug candidates isn’t necessarily their potency. Instead, various other obstacles impede this progress. These include insufficient specificity for PDE5 compared to other PDEs, unfavorable pharmacokinetics, limited in vivo effectiveness, and a lack of well-defined safety profiles for many of the inhibitors reported.

The recent advancements in reporting novel chemical scaffolds as allosteric PDE5-Is with high potency and isozyme selectivity represent an exciting avenue for further research and optimization of their therapeutic benefits. Regardless of the limitations, the balance between benefits and risks highly favors the advantageous utilization of PDE5-Is, and their use continues to rise. Although non-selective targeting of isozymes could result in undesired side effects, the existence of PDE5-Is with dual impacts on factors contributing to the same pathological condition, particularly multi-factorial diseases, could potentially offer an advantage over the traditional single target-one disease approach.

## Figures and Tables

**Table 1:** Summary of the reported cocrystal structures for PDE5 inhibitors and their key interactions with the PDE5 active site.

Scaffold and Compound No.	PDB Code	Cocrystal Structure	Key Ligand Interactions with the PDE5 Active Site
Pyrimidinone, Cpd. **2**	4G2W	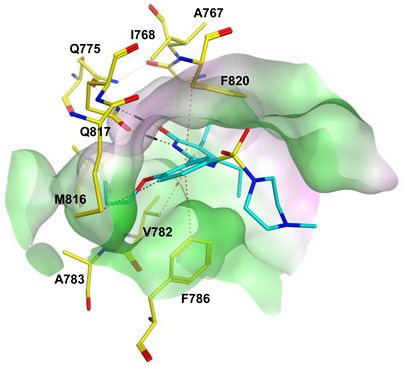 **Sildenafil-like**	The pyrimidinone core is anchored through; a bidentate hydrogen bond with Gln817, CH-pi stacking with Phe820, Val782 and Phe786, in addition to a hydrogen bond with Met816
Pyrimidinone, Cpd. **4**	4I9Z	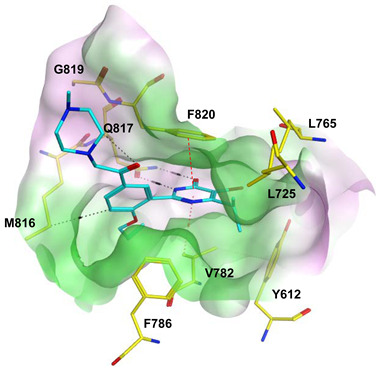 **Sildenafil-like**	The pyrimidinone core is anchored through; a bidentate hydrogen bond with Gln817, π-π stacking with Phe820, a CH-pi interaction with Val782, in addition to a hydrogen bond with Met816
Pyrimidinone, Cpd. **5**	4IAO	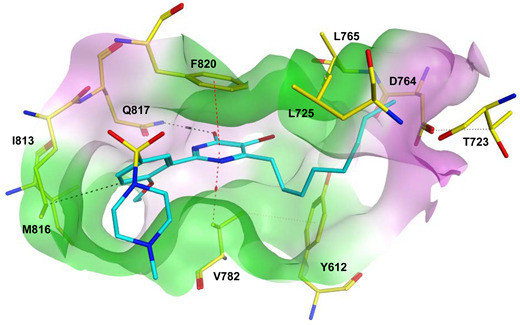 **Tadalafil-like**	The pyrimidinone core is anchored through; a monodentate hydrogen bond with Gln817, π-π stacking with Phe820, a CH-pi interaction with Val782, in addition to a hydrogen bond with Met816
Pyrimidine, Cpd. **9** (Avanafil)	6L6E	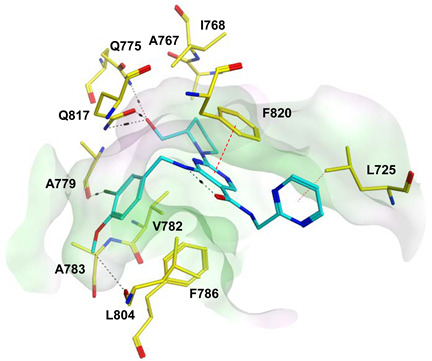 **Sildenafil-like**	The pyrimidine core is anchored through; a bidentate hydrogen bond between the terminal hydroxy group on the pyrrolidine ring and Gln817, π-π stacking with Phe820, in addition to a CH-pi interaction with Leu725, a hydrogen bond with Gln775 and Leu804, as well as a halogen bond with Ala779
Chromenopyrrolones Cpd. **32**	4MD6	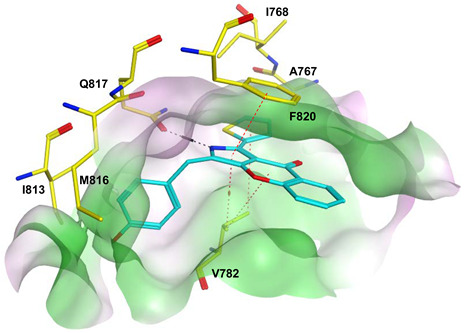 **Tadalafil-like**	The chromenopyrrolone core is anchored through; a monodentate hydrogen bond between the pyrrole ring and Gln817, π-π stacking with Phe820 and three CH-pi interactions with Val782
Chromenopyrrolones, Cpd. **36**	5ZZ2	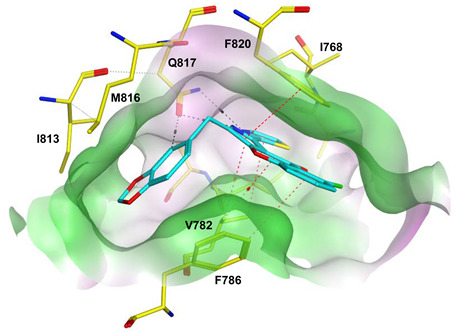 **Sildenafil-like**	The chromenopyrrolone core is anchored through; a bidentate hydrogen bond with Gln817, π-π stacking with Phe820, three CH-pi interactions with Val782, in addition to; a CH-pi interaction with Phe786, and a hydrogen bond between the benzodioxole moiety and Gln817
Azepinoindolone, Cpd. **39**	7FAQ	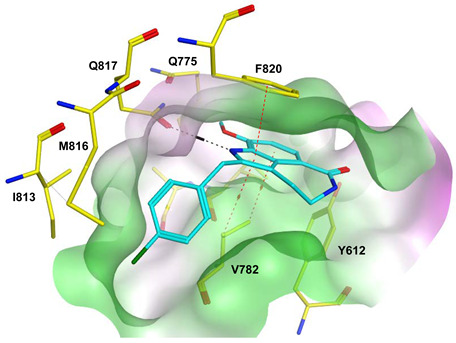 **Tadalafil-like**	The tetrahydro-1H-azepinoindolo-1-one core is anchored through: a monodentate hydrogen bond with Gln817, π-π stacking with Phe820, and two CH-pi interactions with Val782
Azepinoindolone, Cpd. **40**	7FAR	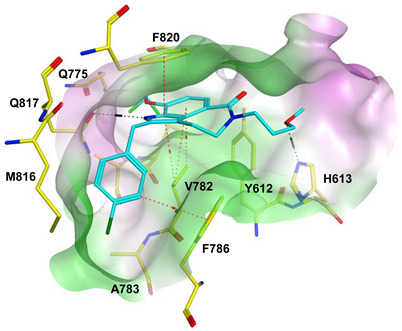 **Tadalafil-like**	The tetrahydro-1H-azepinoindolo-1-one core is anchored through: a monodentate hydrogen bond with Gln817, π-π stacking with Phe820, three CH-pi interactions with Val782, a CH-pi interaction with Phe786 and a hydrogen bond with His613
Indolopyrazinoquinazolinone, Cpd. **46**	6VIB	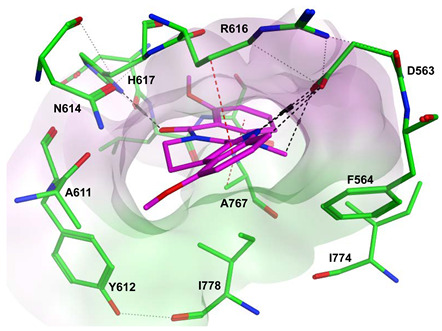 **Allosteric Inhibitor**	The compound is anchored in the allosteric site through; four hydrogen bonds with Asp563, a hydrogen bond with His617 and two CH-pi interactions with Arg616 and Ala767

**Table 2:** Recent clinical trials investigating the effects of PDE5 inhibitors in various diseases.

Identifier	Phase	Intervention	No. of Subjects	Targeted Disease	Main Finding(s)	Ref.
NCT02277132	II/III	Sildenafil 25 mg TID vs. placebo	216 (108 sildenafil and 108 placebo)	Severe early-onset fetal growth restriction in pregnant women	Antenatal maternal sildenafil administration did not reduce the risk of perinatal mortality or major neonatal morbidity. The trial was terminated due to increased risk of neonatal pulmonary hypertension.	[288]
NCT02951429	II	Pirfenidone 801 mg TID + 20 mg TID sildenafil or placebo	177 (88 sildenafil and 89 placebo)	Advanced idiopathic pulmonary fibrosis and risk of pulmonary hypertension	No difference in the proportion of patients with disease progression over 52 weeks between the sildenafil and placebo groups. Similar treatment-emergent adverse events and/or mortality were reported in patients in both groups.	[289]
NCT01178073	III	Tadalafil + ambrisentan (COMB) vs. monotherapy of either agent (MONO)	216 (117 COMB and 99 MONO)	Connective tissue disease-associated pulmonary arterial hypertension and systemic sclerosis-pulmonary arterial hypertension	The risk of clinical failure was lower with COMB vs. MONO (risk reduction: CTD-PAH 51.7%, SSc-PAH 53.7%), particularly in patients with haemodynamic parameters characteristic of typical PAH without signs of left heart disease and/or restrictive lung disease at baseline.	[290]
NCT03238365	Early I	Nivolumab 240 mg IV on days 1 and 15 followed by surgery on day 28 + tadalafil 10 mg orally once daily for 4 weeks or placebo	50	Resectable Head and Neck Squamous Cell Carcinoma	After 4 weeks of treatment, preoperative nivolumab and tadalafil combination was found safe and more than 50% of the patients showed at least 20% treatment response. Posttreatment specimens showed augmentation of the immune microenvironment (B- and natural killer cell gene sets in the tumor and effector T cells in the periphery) with the addition of tadalafil.	[291]
NCT01553721	II	Udenafil 50 mg BID vs. placebo	63	Pulmonary Arterial Hypertension	Udenafil improves exercise capacity among the patients with a history of endothelin receptor antagonist therapy. There were no significant differences in the Borg dyspnea score and time to clinical worsening between groups.	[292]
NCT04489446	I/II	Sildenafil 25 mg orally TID for seven days vs. placebo	40 (20 sildenafil and 20 placebo)	COVID-19 in patients showing perfusion abnormalities	Sildenafil led to significantly shorter median length of hospital stay than the placebo group (9 IQR 7–12 days vs. 12 IQR 9–21 days, *p* = 0.04). No statistically significant differences were found in the oxygenation parameters (PaO_2_/FiO_2_ ratios and A-a gradients).	[293]
NCT01970176	I/II	20 mg tadalafil vs. placebo, repeated after 12 weeks	20 (13 tadalafil and 7 placebo)	Pre-Heart Failure	Long-term tadalafil did not improve glomerular filtration rate (median increase of 2.0 mL/min in the tadalafil group versus 13.5 mL/min in the placebo group; *p* = 0.54). There was no difference in urinary sodium or cGMP excretion with tadalafil following short-term saline loading.	[294]
NCT01484431	I/II	Tadalafil 2.5, 10, 20, or 40 mg orally, once daily	19	Pediatric Patients with Pulmonary Arterial Hypertension	Plasma tadalafil concentrations in pediatric patients aged 2 to <18 years were similar to those in adults at similar doses in a previous trial and confirmed that dosing of 40 mg once daily in pediatric patients with a bodyweight ≥ 40 kg, and a dose of 20 mg once daily in patients with a body weight < 40 kg and aged ≥ 2 years are suitable for phase III evaluation.	[295]
NCT02832570	III	Sildenafil 100 mg oral single dose	14	Arterial claudication	Maximal walking time was significantly improved during the sildenafil period compared with the placebo period (300 s [95% CI 172 s–428 s] vs. 402 s [95% CI 274 s–529 s] *p* < 0.01). Sildenafil had no significant effect on pain-free walking time or skin tissue oxygenation during exercise.	[296]
NCT03049540	III	Tadalafil 20 mg once daily vs. placebo	100	Congenital heart disease with systemic right ventricles	Right ventricular systolic function, exercise capacity and neuro-hormonal activation remained stable over a 3-year follow-up period where no significant treatment effect of tadalafil was observed.	[297]
NCT03566914	II	Tadalafil 10 mg daily vs. placebo for 12 weeks	140 (70 tadalafil and 70 placebo)	Erectile dysfunction in patients with cirrhosis	More patients in tadalafil group achieved > 5 points increase in the erectile function domain of the International Index of Erectile Function when compared with the placebo group [44(62.9%) vs. 21(30%), *p* < 0.001]. Patients receiving tadalafil had significantly more decline in the scores of GAD-7 (assessing anxiety) and PHQ-9 (assessing depression).	[298]
NCT02741115	III	Udenafil 87.5 mg oral BID	386 (191 udenafil and 195 placebo)	Single ventricle heart disease	Udenafil group had significantly improved between baseline and 26 weeks visits compared to placebo group in myocardial performance index (*p* = 0.03), atrioventricular valve inflow peak E (*p* = 0.009), and A velocities (*p* = 0.034), and annular Doppler tissue imaging-derived peak e’ velocity (*p* = 0.008). There were no significant differences in change in single ventricle size, systolic function, atrioventricular valve regurgitation severity, or mean fenestration gradient.	[299]
NCT04283240	Early I	Sildenafil 20 mg single oral dose vs. placebo as add-on to conventional therapy	20 (10 sildenafil and 10 placebo)	Acute intermediate-high risk pulmonary embolism	Sildenafil did not improve cardiac index compared to baseline (0.02 ± 0.36 l/min/m^2^, *p* = 0.89) and neither did placebo (0.00 ± 0.34 l/min/m^2^, *p* = 0.97). Sildenafil lowered mean arterial blood pressure (−19 ± 10 mmHg, *p* < 0.001) which was not observed in the placebo group (0 ± 9 mmHg, *p* = 0.97).	[300]
NCT01720524	III	Sildenafil IV (loading: 0.1 mg/kg, over 30 min; maintenance: 0.03 mg/kg/h) vs. placebo, for 14 days	59 (29 sildenafil and 30 placebo)	Persistent pulmonary hypertension of newborn (receiving iNO)	Treatment failure rates did not differ with sildenafil (27.6%) vs. placebo (20.0%; *p* = 0.4935). Mean time on iNO was not different with sildenafil (4.1 days) vs. placebo (4.1 days; *p* = 0.9850).	[301]
NCT02057458	II	Sildenafil 20 mg TID for 4 weeks	19	Cystic fibrosis	4 weeks of sildenafil improved skeletal muscle O_2_ utilization during exercise to similar values observed in healthy individuals.	[302]

## Data Availability

Data sharing is not applicable.

## Supplementary Materials

The following supporting information can be downloaded at: https://www.mdpi.com/article/10.3390/ph16091266/s1, Table S1: Current on-going clinical trials investigating the effects of PDE5 inhibitors in various diseases.

## Abbreviations

ABC: ATP-binding cassette; Ach: Acetylcholine; AChE: Acetylcholine esterase; AD: Alzheimer’s disease; Ala: Alanine; AMP: Adenosine monophosphate; AMPK: AMP-activated protein kinase; APP: Amyloid precursor protein; Arg-1: Arginase 1; ASCs: Adipose stem cells; ATP: Adenosine triphosphate; BBB: Blood brain barrier; Bcl-2: B-cell lymphoma 2; Bcl-xL: B-cell lymphoma extra-large; BDNF: Brain-derived neurotrophic factor; BID: twice a day; BPH: Benign prostatic hyperplasia; BTB: Blood tumor barrier; cAMP: Cyclic adenosine monophosphate; CDK5: Cyclin-dependent kinase 5; CF: Cystic fibrosis; CFTR: CF transmembrane conductance regulator; cGMP: Cyclic guanosine monophosphate; CNS: Central nervous system; COVID-19: Coronavirus disease of 2019; COX: Cyclooxygenase; CREB: cAMP response element-binding element; CRF-R1: Corticotropin-releasing factor receptor 1; CRPC: Castration-resistant prostate cancer; CVS: Cardiovascular system; DNA: Deoxyribonucleic acid; dox: Doxorubicin; EC_30_: 30% of maximal effective concentration; EC_50_: Half maximal effective concentration; ED: Erectile dysfunction; EMEA: European Medicines Agency; EPC: Endothelial progenitor cell; ER: Endoplasmic reticulum; ERK: Extracellular signal-regulated kinase; FDA: Food and Drug Administration; GABA: Gamma-aminobutyric acid; Gln: Glutamine; GLUT1: Glucose transporter 1; GRP: Glucose regulated protein; GSK3β: Glycogen synthase kinase 3β; HDAC: Histone deacetylase; HEK293: Human embryonic kidney cells 293; HER2: Human epidermal growth factor receptor 2; HF: Heart failure; Hgf: Hepatocyte growth factor; HIF-1α: Hypoxia-inducible factor 1-alpha; His: Histidine; HR: Homologous recombination; HSP90: Heat shock protein 90; I/R: Ischemic and reperfusion; IC_50_: Half maximal inhibitory concentration; IPPS: International Prostate Symptom Score; JNK: Jun N-terminal kinase; Kg: Kilogram; LC_50_: Half maximal lethal concentration; Leu: Leucine; LUTS: Lower urinary tract symptoms; LV: Left ventricular; MAPK: Mitogen-activated protein kinase; MDR: Multidrug resistance; MDSCs: Myeloid-derived suppressor cells; MEKK1: Mitogen-activated protein kinase kinase kinase 1; Met: Methionine; MetS: Metabolic syndrome; mg: Milligrams; MI: Myocardial infraction; mRNA: Messenger ribonucleic acid; MS: Multiple sclerosis; MTDLs: Multitarget-directed ligands; mTOR: Mammalian target of rapamycin; NFAT: Nuclear factor of activated T-cells; NFκB: Nuclear factor-κB; NHEJ: Non-homologous end joining; NIHL: Noise-induced hearing loss; NMDA: N-methyl-D-aspartate; NMDAR: N-methyl-D-aspartate receptors; NO: Nitric oxide; NOS: Nitric oxide synthase; NSAID: Non-steroidal anti-inflammatory drug; NSC: Neural stem cells; PAH: Pulmonary arterial hypertension; PAMPA: Parallel Artificial Membrane Permeability Assay; PARP: Poly (ADP-ribose) polymerase; PBMCs: Peripheral blood mononuclear cells; PBS: Phosphate buffer saline; PCSCs: PC3-derived cancer stem cells; PDE: Phosphodiesterase; PDE5-Is: Phosphodiesterase 5 inhibitors; PGC-1α: Peroxisome proliferator-activated receptor gamma coactivator 1-alpha: Phe: Phenylalanine; PI3K: Phosphoinositide 3-kinase; PK: Pharmacokinetic; PKC: Protein kinase C; PKG: Protein kinase G; PS1: Presenilin-1; R&D: Research and development; ROS: Reactive oxygen species; SAR: Structure activity relationship; sGC: Soluble guanylyl cyclase; SI: Selectivity index; Sirt3: Sirtuin-3; SLE: Systemic lupus erythematosus; SMR: Smooth muscle relaxation; SOSA: Selective optimization of side activities; SS: Sulindac sulfide; SSc: Scleroderma; SSRIs: Selective serotonin reuptake inhibitors; TAZ: Tafazzin; TID: three times daily; TGF-β1: Transforming growth factor beta 1; THβC: Tetrahydro-beta-carbolines; TNF: Tumor necrosis factor; Tyr: Tyrosine; Val: Valine; VEGFA: Vascular endothelial growth factor A; Vegfa: Vascular endothelial growth factor; ZBG: Zinc binding group.
