# Supplementary material for: Advancements in Phosphodiesterase 5 Inhibitors: Unveiling Present and Future Perspectives
Source: Pharmaceuticals (Basel). 2023 Sep 6;16(9):1266. doi: 10.3390/ph16091266 (PMC10536424; doi:10.3390/ph16091266)
Supplement: Supplementary file 1 [file pharmaceuticals-16-01266-s001.zip › pharmaceuticals-2513577-supplementary.pdf]

Supplementary Material

Advancements in Phosphodiesterase 5 Inhibitors: Unveiling Present and Future Perspectives

[1]School of Life and Medical Sciences, University of Hertfordshire Hosted by Global Academic Foundation. New Administrative Capital, Cairo 11865, Egypt.

[2]Department of Chemistry, Faculty of Pharmacy, Ahram Canadian University, Cairo, 12451, Egypt.

[3]Department of Pharmaceutical Chemistry, Faculty of Pharmacy and Biotechnology, German University in Cairo, Cairo 11835, Egypt.

#Authors equally contributed to this work
*Correspondence: ashraf.abadi@guc.edu.eg , Tel +201001588454;

Table S1: Current on-going clinical trials investigating the effects of PDE5 inhibitors in various diseases

Identifier	Phase	Intervention	Targeted disease	Primary outcome measure(s)
NCT04684589	II	Tadalafil 20 mg vs placebo	Obesity	Thermoneutral FSF of WAT at the level of the umbilicus at 12 Weeks., MRI measurement of lipid content, 12 weeks. Subcutaneous adipose tissue expression of UCP1 (normalized expression units), Measurement of gene expression in adipose sample, 12 weeks.
NCT04039087	II/III	Sildenafil 40 mg vs placebo	Cystic Fibrosis	6 Minute Walk Distance (6MWD), capacity, an objective measurement of exercise tolerance, predicts mortality in patients with CF. Change in distance walked between week 1 and week 12.
NCT05201144	II	Sildenafil oral suspension vs placebo	Congenital Diaphragmatic Hernia Pulmonary Hypertension	Change in Left Ventricular Eccentricity Index (LVEI) on echocardiogram after 14 days of study treatment compared to baseline echocardiogram as compared to placebo.
NCT05951413	II/III	Sildenafil Citrate + estradiol	IVF	Endometrial thickness, measured by transvaginal ultrasound, 10 to 16 days from start of cycle.
NCT05844462	III	Tadalafil vs placebo	Pulmonary Hypertension Chronic Obstructive Pulmonary Disease	6-minute Walk; the distance covered in meter during a 6-minute walk at week 16 post-randomization for patients treated with tadalafil compared to placebo, 16 weeks
NCT05061368	II	Sildenafil Citrate vs placebo	Chronic Obstructive Pulmonary Disease	Exercise Capacity and maximal oxygen uptake (peak VO_2), within 20-25 minutes post-dose.
NCT05709574	II	Tadalafil 20 mg	Gastric Adenocarcinoma Gastroesophageal Junction Adenocarcinoma	Evaluating the safety and tolerability of tadalafil treatment with FLOT chemotherapy
NCT05558176	IV	Sildenafil citrate	Fetal Hypoxia	Incidence of non-reassuring fetal heart rate patterns; a non-reassuring fetal heart rate pattern will be defined as one or more of the following features; 60 Bradycardia, FHR <110 bpm Tachycardia, FHR > 160bpm Baseline variability < 5 bpm for 30 to 50 minutes OR more than 25 bpm for 15 to 25 minutes.
NCT05556083	II	Dapoxitine 3 mg + Sildenafil 50 mg or placebo	Premature Ejaculation	Intravaginal latency time changes IVLT using PEDT premature ejaculation diagnostic tool (PEDT), eight weeks for each participant of both groups. Overall partner satisfaction, using Sexual Satisfaction Index (SSI), eight weaks for each participant of both groups
NCT05490680	III	Sildenafil oral film 25 mg, 50 mg, 75 mg or 100 mg vs placebo	Erectile Dysfunction	Safety of Sildenafil doses versus placebo, Safety of Sildenafil doses versus placebo, i.e., the proportion of subjects with at least one Treatment Emergent Adverse Events (TEAEs) of Special Interest ("Headache" or "Dizziness"), 12 weeks of treatment<br>EEfficacy of Sildenafil oral film versus placebo evaluated using co-primary efficacy endpoint from the change in Erectile Function (EF) domain of the

				International Index for Erectile Function (IIEF) questionnaire, baseline to the end of the 12-week treatment period
NCT05275725	I	Sildenafil	Birth Asphyxia	Maximum tolerable dose of sildenafil, within 30 days of drug administration
NCT05266651	II/III	Tadalafil 5 mg vs placebo	Sexual Dysfunction	Clitorial Artery Doppler, Pulsatile index, Resistance index and Peak systolic velocity after one month duration of drug use.
NCT05206955	III	Tadalafil vs placebo	Fontan Palliation	Microvascular endothelial function measured by the reactive hyperemic index using peripheral artery tonometry (EndoPAT), 52 weeks.<br>Pulmonary vascular reserve; the slope of mean pulmonary artery pressure / cardiac output (mPAP/CO), 52 weeks.
NCT05195775	II/III	Tadalafil	Duchenne Muscular Dystrophy	Change in post-contractile BOLD response after tadalafil dosing. MRI technique to measure microvascular function in skeletal muscle, done 3 hours after dosing/no-dosing on two separate study visits.<br>Change in post-exercise hyperemia after tadalafil dosing using Doppler ultrasound done 3 hours after dosing/no-dosing on two separate study visits.
NCT05173896	II	Tadalafil 20 mg vs placebo	Cerebral Small Vessel Diseases<br>Stroke, Ischemic	Feasibility of treatment defined as proportion of participants achieving full target dose of tadalafil/placebo., Number of participants achieving full target dose of tadalafil/placebo by end of three months trial period.
NCT05030623	I/ II	Tadalafil 5 mg vs placebo	Major Depressive Disorder	Effect on Hamilton Depression rating scale score (HAM-D score), Remission is defined as HAM-D total score 7 (primary outcome). Treatment response is defined as 50% drop in the HAM-D total score., 12 weeks.
NCT04908657	IV	Sildenafil 20 mg	Liver Fibrosis	Liver MRI, Intra-voxel incoherent motion (IVIM) imaging, from baseline to 3 years.
NCT04797286	II	Sildenafil vs placebo	Scleroderma<br>Mildly Elevated Pulmonary Pressures	Difference in change in distance walked in 6-minute walk test (6MWT) at 4 months.
NCT04565925	II	Sildenafil Citrate vs placebo	Spinal Cord Injuries<br>Urinary Incontinence	Bladder Leakage as measured by 5-day bladder diary, which will be kept for five 24 hours periods for 4 weeks of sildenafil treatment
NCT04538976	IV	Sildenafil	Acute Exacerbation of COPD	Time alive and out of hospital, 365 days
NCT04447989	II	Sildenafil vs placebo	Bronchopulmonary Dysplasia of Newborn	Safety as determined by incidence of hypotension experienced by the participants through 28 days post last dose of study drug.
NCT04356716	II	Sildenafil	Choroidal Ischemia - Age-related Macular Degeneration - Central Serous Retinopathy - Retinitis Pigmentosa	Change in Choroidal Perfusion; patients are evaluated for progression of disease/dystrophy via visual acuity (improved/stable/worsened) and appearance of fluid layer and drusen on OCT testing, up to 5 Years
NCT03686306	III	Sildenafil vs placebo	Peripheral Artery Disease	Absolute change of the absolute claudication distance (ACD) from baseline to week 24; Baseline and week 24
NCT02682147	IV	Sildenafil	Emphysema	Perfused blood volume assessed by CT scan at two time points and compared at two points, pre and post the administration of sildenafil.

NCT04039464	III	Mono-Therapy with Sildenafil vs combination with Bosentan	Pediatric Pulmonary Hypertension	Change in WHO functional class of pulmonary hypertension per participant in each arm (Mono vs. Dual Therapy); Baseline, 12 months.
NCT05014776	II	Tadalafil + Pembrolizumab + Ipilimumab	Pancreatic Cancer	Objective response rate (irORR) using immune Response Evaluation Criteria for Solid Tumors (iRECIST); irORR is defined as the number of patients achieving a complete response (irCR) or partial response (irPR) based on the immune Response, 4 years.
NCT05051436	IV	Mirabegron 50 mg + Tadalafil 10 mg or placebo	Pre-diabetes<br>Obesity	Oral glucose tolerance test using 75 g of glucose at baseline and at 14 weeks and blood glucose will be measured.
NCT05183334	IV	Tramadol hydrochloride 100 mg + Sildenafil 50 mg or placebo	Premature Ejaculation	Score of sexual satisfaction, 8 weeks.
